# Characterizing Genetic Regulatory Elements in Ovine Tissues

**DOI:** 10.3389/fgene.2021.628849

**Published:** 2021-05-20

**Authors:** Kimberly M. Davenport, Alisha T. Massa, Suraj Bhattarai, Stephanie D. McKay, Michelle R. Mousel, Maria K. Herndon, Stephen N. White, Noelle E. Cockett, Timothy P. L. Smith, Brenda M. Murdoch, on behalf of The Ovine FAANG Project Consortium

**Affiliations:** ^1^Department of Animal, Veterinary, and Food Science, University of Idaho, Moscow, ID, United States; ^2^Department of Veterinary Microbiology and Pathology, Washington State University, Pullman, WA, United States; ^3^University of Vermont, Burlington, VT, United States; ^4^USDA, ARS, Animal Disease Research Unit, Pullman, WA, United States; ^5^Paul G. Allen School for Global Animal Health, Washington State University, Pullman, WA, United States; ^6^Center for Reproductive Biology, Washington State University, Pullman, WA, United States; ^7^Utah State University, Logan, UT, United States; ^8^USDA, ARS, U.S. Meat Animal Research Center (USMARC), Clay Center, NE, United States

**Keywords:** FAANG, epigenetics, ChIP-seq, WGBS, methylation, sheep, functional genomics, histone modifications

## Abstract

The Ovine Functional Annotation of Animal Genomes (FAANG) project, part of the broader livestock species FAANG initiative, aims to identify and characterize gene regulatory elements in domestic sheep. Regulatory element annotation is essential for identifying genetic variants that affect health and production traits in this important agricultural species, as greater than 90% of variants underlying genetic effects are estimated to lie outside of transcribed regions. Histone modifications that distinguish active or repressed chromatin states, CTCF binding, and DNA methylation were used to characterize regulatory elements in liver, spleen, and cerebellum tissues from four yearling sheep. Chromatin immunoprecipitation with sequencing (ChIP-seq) was performed for H3K4me3, H3K27ac, H3K4me1, H3K27me3, and CTCF. Nine chromatin states including active promoters, active enhancers, poised enhancers, repressed enhancers, and insulators were characterized in each tissue using ChromHMM. Whole-genome bisulfite sequencing (WGBS) was performed to determine the complement of whole-genome DNA methylation with the ChIP-seq data. Hypermethylated and hypomethylated regions were identified across tissues, and these locations were compared with chromatin states to better distinguish and validate regulatory elements in these tissues. Interestingly, chromatin states with the poised enhancer mark H3K4me1 in the spleen and cerebellum and CTCF in the liver displayed the greatest number of hypermethylated sites. Not surprisingly, active enhancers in the liver and spleen, and promoters in the cerebellum, displayed the greatest number of hypomethylated sites. Overall, chromatin states defined by histone marks and CTCF occupied approximately 22% of the genome in all three tissues. Furthermore, the liver and spleen displayed in common the greatest percent of active promoter (65%) and active enhancer (81%) states, and the liver and cerebellum displayed in common the greatest percent of poised enhancer (53%), repressed enhancer (68%), hypermethylated sites (75%), and hypomethylated sites (73%). In addition, both known and *de novo* CTCF-binding motifs were identified in all three tissues, with the highest number of unique motifs identified in the cerebellum. In summary, this study has identified the regulatory regions of genes in three tissues that play key roles in defining health and economically important traits and has set the precedent for the characterization of regulatory elements in ovine tissues using the Rambouillet reference genome.

## Introduction

Regulatory element characterization and chromatin state determination in relevant tissues was identified as a critical need for implementing precision breeding within the livestock industry by the Agricultural Animal Genomics Community (Rexroad et al., 2019). To this end, the Functional Annotation of Animal Genomes (FAANG) consortium and the Ovine FAANG project members seek to molecularly define the epigenome in food animals, including sheep (Andersson et al., 2015; Tuggle et al., 2016; Giuffra et al., 2019). Modeled upon the ENCODE project (The ENCODE Project Consortium, 2012), FAANG aims to characterize the epigenome including chromatin histone modifications and DNA methylation (Andersson et al., 2015). The core objective of the Ovine FAANG Project Consortium is to develop a deep and robust public database of transcriptional regulatory features in the sheep genome.

Sheep production for meat, milk, and wool is an important agricultural industry across the globe with more than one billion sheep suited to a diverse range of climates (Hegde, 2019). This diversity is reflected in genetic differences between sheep breeds utilized for varied purposes (Meadows et al., 2008; Al-Mamun et al., 2015). Populations bred for different environments and for contrasting production traits provide the opportunity to study a range of phenotypes within the species. Analysis of elements that control gene expression in sheep tissues is needed as many complex traits such as rumen fatty acid metabolism, lanolin and wool production, growth, and carcass traits cannot be explained solely by variation in transcribed regions (Jiang et al., 2014; Villar et al., 2015; Clark et al., 2017; Kingsley et al., 2019). *In vivo* analysis of regulatory elements will allow researchers to test hypotheses of biological function of putative causal mutations in relevant production tissues. Understanding the phenotypic influences of genetic variance that lie in promoter and enhancer regions is important for trait prediction and the improvement of sheep production.

Functional variants that are causally implicated in phenotypic variation are increasingly found to lie outside of transcribed regions within DNA regulatory elements (Albert and Kruglyak, 2015; Xiang et al., 2019). These regulatory elements can be defined by epigenetic analyses that have not been systematically conducted in sheep. A library of putative regulatory elements in the sheep genome was recently predicted using inference from chromatin states defined in humans (Naval-Sanchez et al., 2018). However, direct experimental characterization of regulatory elements in individual ovine tissues is needed.

The work presented here represents the foundation in the preparation for a deep survey using the same methodology across tissues of the index animal from which the new sheep reference genome was developed. Since the larger FAANG effort has *N* = 2 for each tissue by design (i.e., a large array of tissues from the individual from which the genome was derived), the data collected here also provide a resource for evaluating the larger effort by permitting estimation of interindividual variation in the appearance and tissue distribution of regulatory elements. Three tissues were selected for this study based on their prominence in defining production traits and to span tissues of endodermal, mesodermal, and ectodermal origin and because each presents unique procedural challenges for performing chromatin immunoprecipitation with sequencing (ChIP-seq) assays. The liver is an endodermal-derived tissue that is a key metabolic component of the alimentary system (Villar et al., 2015) and contains a variety of complex carbohydrates that can inhibit various enzymatic reactions required in the ChIP-seq protocol. The spleen is a mesodermal-derived parenchymatous organ important for immune cell production and maturation and contains many natural deoxyribonucleases (DNase) which can present challenges to obtaining sufficient yield of high-quality DNA (Young and Sinsheimer, 1965). The cerebellum is an ectodermal-derived tissue representative of brain tissue and contains a high lipid content which can affect the efficiency of DNA extraction. With these three varied tissues, we developed workflows for assessing chromatin-associated histone modifications, CTCF-binding sites, and DNA methylation to define regulatory elements.

The histone modifications characterized in this study include the trimethylation of histone 3 lysine 4 (H3K4me3) which denotes promoters and acetylation of histone 3 lysine 27 (H3K27ac) which denotes active enhancers (Barski et al., 2007; Wang et al., 2008). The monomethylation of histone 3 lysine 4 (H3K4me1) was characterized to explore poised enhancers, and the trimethylation of histone 3 lysine 27 (H3K27me3) was utilized to define repressed enhancers which silences gene expression in broad regions (Barski et al., 2007; Wang et al., 2008; Pauler et al., 2009). The CCTC-binding factor protein (CTCF) is a key component of the anchors at topologically associated domain boundaries (Lee and Iyer, 2012; Ghirlando and Felsenfeld, 2016). Determination of CTCF and multiple histone modifications, referred to as marks, allowed us to take advantage of the combinatorial nature of chromatin structure and gene expression regulation (Jenuwein and Allis, 2001; Wang et al., 2008) to categorize the sheep genome into chromatin states.

DNA methylation data derived from whole-genome bisulfite sequencing (WGBS) were incorporated to validate regulatory regions and chromatin states. In mammals, several groups have identified CpG islands that lack methylation are located at gene promoters (Deaton and Bird, 2011). Repressed promoters are marked by higher degrees of methylation associated with transcriptionally silenced gene expression (Weber et al., 2007). Histone methylation and DNA methylation are co-dependent epigenetic marks as enzymatic formation of one will guide the formation of the other and H3K4me3 may physically inhibit methylation of DNA during development (Meissner et al., 2008). Histone methylations and DNA methylation serve as templates for rebuilding one another during mitosis and meiosis and further reinforce segmentation of the genome into functional regions of active or repressed chromatin in adult somatic cells (Cedar and Bergman, 2009) justifying the utility of combined analysis in sheep.

Our objective for this study was to identify the locations of gene regulatory elements in sheep by characterizing histone modifications, CTCF binding, and DNA methylation for the cerebellum, liver, and spleen. Defining regulatory elements in the sheep genome will provide the basis for a greater understanding of the mechanisms that underpin phenotypic variation in important health and production traits in sheep.

## Materials and Methods

### Sample Collection

Tissue was collected postmortem from two pairs of healthy half siblings (one ewe and one wether per pair) totaling four yearling crossbred sheep (Columbia, Polypay, Rambouillet, Suffolk, Targhee) as approved by the Washington State University Institutional Animal Care and Use Committee. Small pieces of liver, spleen, and cerebellum tissues were collected within 30 min postmortem, briefly rinsed with ice cold 1 × PBS, and promptly snap frozen in liquid nitrogen. Samples were transferred from liquid nitrogen directly into a −80°C freezer for storage.

### Chromatin Immunoprecipitation

Chromatin immunoprecipitation (ChIP) was performed using commercial antibodies for the histone modifications H3K4me3 (Abcam, cat. # ab8580), H3K4me1 (Abcam, cat. # ab8895), H3K27ac (Abcam, cat. # ab4729), H3K27me3 (Abcam, cat. # ab6002), and CTCF (Millipore, cat. # 07-729) with SimpleChIP Plus Enzymatic Chromatin IP Kit according to the manufacturer’s instructions (Cell Signaling Technologies cat. # 9005, Danvers, MA, United States) (Barski et al., 2007; Johnson et al., 2007; Mikkelsen et al., 2007; Robertson et al., 2007; Park, 2009). Briefly, tissue was cross-linked with 37% formaldehyde and disaggregated with a Dounce homogenizer. After cell membrane lysis, micrococcal nuclease (MNase) was added and incubated at 37°C and 200 rpm for 20 min to shear the chromatin. Next, the nuclear membrane was lysed, and the sheared chromatin isolated by centrifuging at 15,000×*g* for 1 min at 4°C. Chromatin was incubated with 1 μg of antibody overnight at 4°C in a Hula mixer for 16 h. The following morning, protein G-coated magnetic beads were added and incubated 2 h at 4°C in the Hula mixer. The sample was washed twice with a low salt and once with a high salt buffer. Cross-links were reversed by incubating the sample at 65°C for 30 min at 400 rpm in a thermomixer. Purification was performed with the DNA Purification Buffers and Spin Columns Kit following the manufacturer’s instructions (Cell Signaling Technologies, cat. # 14209, Danvers, MA, United States).

### Chromatin Immunoprecipitation With Sequencing Library Preparation and Sequencing

Purified DNA samples were quantified using the Qubit dsDNA HS Assay Kit (Thermo Fisher Scientific, catalog number Q32854, Waltham, MA, United States). The DNA size and integrity were verified using a Fragment Analyzer (Agilent, Santa Clara, CA, United States). Libraries were prepared with the TruSeq ChIP Library Preparation Kit (Illumina, Inc., catalog number IP-202-1012, San Diego, CA, United States) for 75 base pair paired-end reads following the manufacturer’s instructions and sequenced to at least 20 million mapped reads for “narrow” histone marks H3K4me3, H3K27ac, and CTCF libraries and at least 40 million mappable reads each for “broad” histone marks H3K4me1 and H3K27me3 libraries.

### Whole-Genome Bisulfite Sequencing Library Preparation and Sequencing

Whole-genome bisulfite sequencing was performed as a service by Novogene (Beijing, China) on the liver, spleen, and cerebellum in all four animals. Briefly, DNA extracted from these tissues was subjected to agarose gel electrophoresis to test for DNA degradation and potential RNA contamination. The DNA was then quantified using a Nanodrop spectrophotometer (NanoDrop Technologies, Rockland, DE, United States) and a Qubit2.0 fluorometer (Life Technologies, Carlsbad, CA, United States). Lambda phage DNA was spiked in as a negative control for DNA methylation. Since lambda phage DNA lacks DNA methylation, all the cytosines in its DNA should be converted to uracil during bisulfite conversion. Any unchanged cytosine in the lambda phage DNA can thus be used to determine the efficiency of bisulfite conversion. For library construction, DNA samples were fragmented into 200–400 bp using sonication (Covaris S220, Woburn, MA, United States). Next, end repair, A-ligation, and methylation sequencing adapter ligation was performed. The adapter sequences were 5′ adapter (5′-AATGATACGGCGACCACCGAGATCTACACTCTTTCCCTA CACGACGCTCTTCCGATCT-3′) and 3′ adapter (5′-GATC GGAAGAGCACACGTCTGAACTCCAGTCACATCACGATC TCGTATGCCGTCTTCTGCTTG-3′). Following this, the DNA library was subjected to bisulfite treatment (EZ DNA Methylation Gold Kit, Zymo Research, Irvine, CA, United States). Library concentration was first quantified by Qubit2.0, diluted to 1 ng/μl before checking insert size on Agilent 2100 (Agilent Technologies, Santa Clara, CA, United States), and quantified with more accuracy by quantitative PCR (effective concentration of library > 2 nM). Libraries were then pooled per sample and sequenced paired-end.

### Chromatin Immunoprecipitation With Sequencing Data Quality Control, Mapping, and Peak Calling

Quality control assessment of ChIP-seq reads was performed with FastQC, and Trim Galore was used to trim adapter sequences and low-quality bases. PCR duplicates were removed with Picard and the remaining read pair sequences were then mapped to the sheep reference genome *Oar_rambouillet_v1.0* with Bowtie2 (Langmead and Salzberg, 2012; Broad Institute, 2019). Cross-correlations were calculated using MACS2 *predicted* in Galaxy Version 2.1.1.20160309.1 (Supplementary Figure 1) (Afgan et al., 2018). Peaks for narrow histone marks H3K4me3 and H3K27ac as well as transcription factor CTCF were called using MACS2 with an input control and a false discovery rate of 0.05 (Zhang et al., 2008; Feng et al., 2012; Thomas et al., 2017). For broad peak histone modifications H3K4me1 and H3K27me3, SICER was implemented with the same input control and a false discovery rate of 0.05 to better account for broader sequence pileup distributions (Zang et al., 2009; Micsinai et al., 2012; Siska and Kechris, 2017). The number of uniquely mapped sequences, non-redundant fraction (NRF), and fraction of reads in peaks (FRiP) for each ChIP-seq sample were calculated using Picard (Heinz et al., 2010; Friedman and Alm, 2012; Landt et al., 2012; Siska and Kechris, 2017; Afgan et al., 2018) (Supplementary Table 1). Peak numbers were averaged across samples. Peaks common to multiple samples were determined with BEDTools intersect. The peaks common to three samples with the greatest NRF were determined for H3K4me3 (F1, M1, and M2 for liver; F2, M1, and M2 for spleen; and F1, M1, and M2 for cerebellum), H3K27ac (F1, M1, and M2 for liver; F2, M1, and M2 for spleen; and F1, M1, and M2 for cerebellum), H3K4me1 (F1, M1, and M2 for liver; F2, M1, and M2 for spleen; and F1, M1, and M2 for cerebellum), H3K27me3 (F1, M1, and M2 for liver; F2, M1, and M2 for spleen; and F2, M1, and M2 for cerebellum), and CTCF (F2, M1, and M2 for liver; F2, M1, and M2 for spleen; and F2, M1, and M2 for cerebellum). These consensus peaks were compared with transcription start site locations identified with CAGE assays from the ewe used to generate the reference genome using the deepTools *computeMatrix* function, and heatmaps were plotted with the *plotHeatmap* function (Ramírez et al., 2014; Salavati et al., 2020). Furthermore, peaks were annotated with the GTF file from the reference genome *Oar_rambouillet_v1.0*, and peaks were categorized as near a transcription start site (TSS) (+2 to −2 kb), exonic, intronic, near a transcription termination site (TTS) (+1 to −1 kb), and intergenic using the Homer *annotatePeaks.pl* function (Heinz et al., 2010). Furthermore, normalized bigwig files depicting the sequence enrichment for each library were directly visualized with integrative genomics viewer (IGV) for some gene regions which are known to be active and repressed in each tissue (Robinson et al., 2011). Spearman correlations were calculated between sample BAM signal files using deepTools in Galaxy Version 2.1.1.20160309.1 (Friedman and Alm, 2012; Ramírez et al., 2014; Siska and Kechris, 2017; Afgan et al., 2018).

### DNA Methylation Data Quality Control, Mapping, and Methylation Level Characterization

The quality of raw sequences from WGBS was assessed using FastQC v0.11.5. Adapters and low-quality bases (phred score < 20) were trimmed using Trimgalore v0.4.5 with default parameters. Cleaned data for each sample was aligned to the sheep reference genome *Oar_rambouillet_v1.0* using Bowtie2 aligner within BSseeker2 v2.1.8 with default parameters (Langmead and Salzberg, 2012; Guo et al., 2013). The X-chromosome was removed from the analysis to make male and female samples comparable. After mapping, BAM files for the same individual sequenced on multiple lanes were merged, fixmated, and sorted and PCR duplicates were removed using Samtools v1.6 (Li et al., 2009). The methylation level in each cytosine was determined using BSseeker2 with default parameters. Basic statistics on methylation were determined using the *mstat* function in CGmaptools v0.0.6 (Guo et al., 2018). Regions of the genome hypomethylated and hypermethylated for each sample were determined with methPipe v3.4.3 following the manual with default parameters (Song et al., 2013).

### Chromatin State and CTCF Motif Analysis

Chromatin states were characterized by employing a hidden Markov model in ChromHMM, which assessed signal overlap between histone marks within a tissue and binned the genome into a given number of chromatin states (Ernst and Kellis, 2010, 2012, 2017; Gorkin et al., 2017, 2020). The two male samples (M1 and M2) exhibited the greatest NRF and Spearman correlations and were therefore used in chromatin state analysis. The *LearnModel* function in ChromHMM was implemented with given chromatin states of two through 20 for each animal, and the model with the optimal number of chromatin states was examined using the *CompareModels* function in ChromHMM (Gorkin et al., 2017, 2020). The optimal number of chromatin states was determined as the model where the median Pearson correlation for all states plotted against each chromatin state model plateaued and were tightly correlated with the model with the greatest number of states (Supplementary Figure 2) (Gorkin et al., 2017, 2020). The consensus of chromatin states between two animals (M1 and M2) was used to generate the heatmap and for further comparative analyses. Location similarities and differences between chromatin states, hypermethylated regions, and hypomethylated regions were assessed with BEDTools intersect within each tissue, and the consensus within each tissue was used to examine chromatin state and DNA methylation similarities and differences between liver, spleen, and cerebellum tissues (Quinlan, 2014). An Upset R plot was generated to display chromatin state similarities and differences between tissues (Lex et al., 2014; Conway et al., 2017). Significantly enriched known and *de novo* CTCF motifs were identified and compared with other species by implementing the findMotifs.pl script in HOMER (Heinz et al., 2010). The proximity of annotated TSS generated from CAGE data to promoter chromatin states was examined with deepTools *computeMatrix* and *plotHeatmap* functions (Supplementary Figure 7) (Ramírez et al., 2014; Salavati et al., 2020).

## Results

Genetic regulatory elements were characterized across the sheep genome in the liver, spleen, and cerebellum using CTCF binding and ChIP-seq of four histone marks, as well as DNA methylation status. Locating regulatory elements within and between tissues will provide the basis for identifying variation in these elements that may influence various phenotypic traits in sheep. Furthermore, these results represent a resource for estimating interindividual variation in the regulatory states of tissues to provide context for the FAANG project that aims to characterize these states in a broad array of tissues in a single individual from which the reference genome was produced.

### Mapping Summary and Statistics

Mapping statistics were calculated to assess the assay quality, library preparation, and sequence coverage for each sample. Across animals, ChIP-seq reads had a consistent average mapping rate of 78.23, 78.39, and 76.82% to the *Oar_rambouillet_v1.0* genome for the liver, spleen, and cerebellum, respectively. The number of uniquely mapped paired-end reads averaged 40,757,252 for H3K4me3, 42,306,275 for H3K27ac, 53,171,657 for H3K4me1, 55,901,184 for H3K27me3, and 45,491,017 for CTCF across all three tissues. The number of uniquely mapped reads, NRF, and FRiP for each sample are displayed in Supplementary Table 1.

Whole-genome bisulfite sequencing of cerebellum, liver, and spleen samples from the four sheep generated a total of 986, 1,070, and 904 million paired end reads, respectively, with a read length of 2 × 150 bp. The number of reads uniquely mapped to the reference genome was 84.24, 78.86, and 82.48% for the cerebellum, liver and spleen, respectively. The uniquely mapped bases covered the reference genome (*Oar_rambouillet_v1.0*; genome size ∼2.87 Gb) at an average depth of 21 × (range 18× to 26×). Bisulfite conversion rate was ∼99.9% for all the samples. Mapping statistics for each tissue sample per sheep are displayed in Supplementary Table 2.

### Chromatin Immunoprecipitation With Sequencing Peak Calling

**FIGURE 1:**
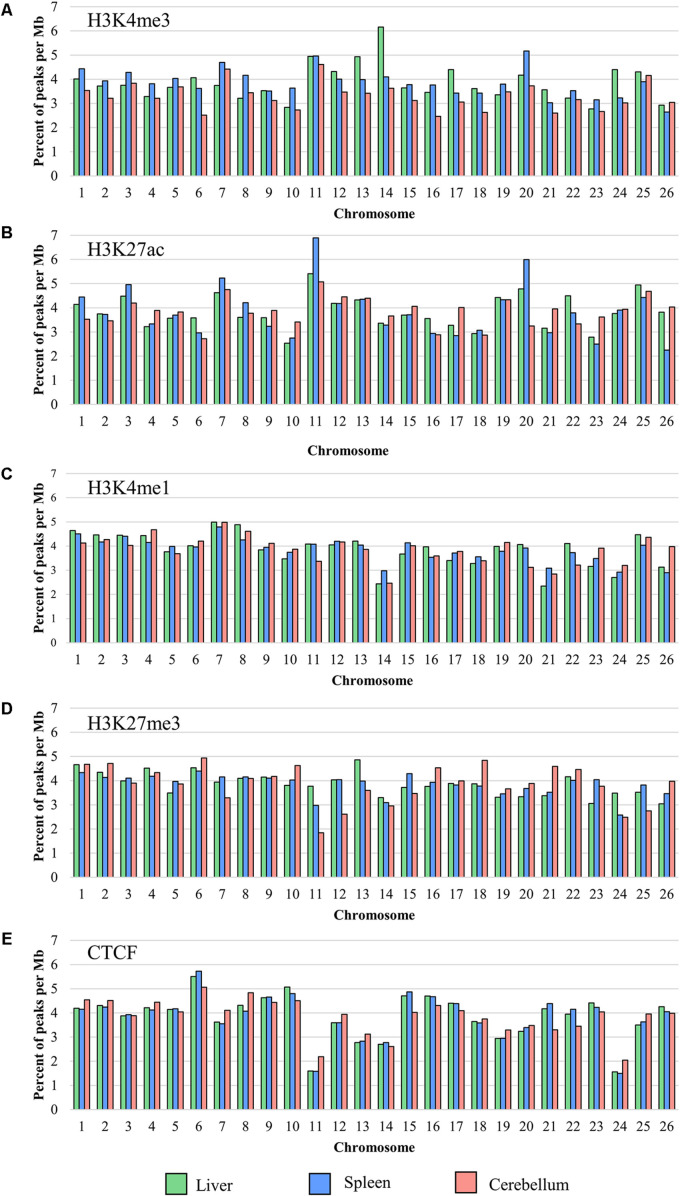
The percent of the total number of peaks normalized per Mb on each chromosome for **(A)** H3K4me3, **(B)** H3K27ac, **(C)** H3K4me1, **(D)** H3K27me3, and **(E)** CTCF averaged from all four animals (F1, F2, M1, and M2).

The locations of sequence signal enrichment were identified for all four histone marks and CTCF for each liver, spleen, and cerebellum sample by mapping the reads to the reference genome *Oar_rambouillet_v1.0*. The number of peaks normalized by chromosome length (in Mb; Figure 1) and the width of the peaks along the assembly were calculated from the mapped read depth. For each mark, the percent of the total number of peaks observed in the genome that lie on each chromosome is plotted in Figure 1 which shows an overall even distribution across chromosomes with some exceptions. The lowest number of peaks was called in narrow mark H3K4me3 (means of 10,458 in the liver, 13,389 in the spleen, and 16,911 in the cerebellum), with the lowest number of peaks per Mb on chromosomes 23 (2.77 peaks/Mb), 26 (2.64 peaks/Mb), and 16 (2.47 peaks/Mb) in the liver, spleen, and cerebellum, respectively. The greatest number of H3K4me3 peaks per Mb for the liver, spleen, and cerebellum was on chromosomes 14 (6.16 peaks/Mb), 20 (5.17 peaks/Mb), and 11 (4.61 peaks/Mb), respectively. The average widths of H3K4me3 peaks were 168, 178, and 313 bp for the liver, spleen, and cerebellum. The mean number of peaks called for the H3K27ac mark was 30,553 in the liver, 35,327 in the spleen, and 35,877 in the cerebellum with the lowest number of peaks called on chromosomes 10 (2.54 peaks/Mb), 26 (2.25 peaks/Mb), and 6 (2.72 peaks/Mb) for the respective tissues. The greatest number of H3K27ac peaks was called on chromosome 11 for all three tissues, and peak widths averaged 239, 240, and 238 bp in the liver, spleen, and cerebellum for this narrow mark. The final narrow mark, CTCF, averaged 26,517 peaks in the liver, 28,362 in the spleen, and 26,244 in the cerebellum. The lowest number of CTCF peaks were called on chromosome 24 (1.56 peaks/Mb for the liver, 1.49 peaks/Mb for the spleen, and 2.05 peaks/Mb in the cerebellum), and the greatest number of peaks were called on chromosome 6 (5.50 peaks/Mb in the liver, 5.73 peaks/Mb in the spleen, and 5.07 peaks/Mb in the cerebellum) for all three tissues. The width of CTCF peaks was similar to other narrow marks, with averages of 114 bp in the liver, 265 bp in the spleen, and 144 bp in the cerebellum.

The greatest number of peaks was called in broad mark H3K4me1 (means of 47,828 in the liver, 33,931 in the spleen, and 51,766 in the cerebellum), which is consistent with several tissues in cattle (Fang et al., 2019). Chromosomes with the lowest number of H3K4me1 peaks per Mb included 21 (2.34 peaks/Mb) for the liver, 26 (2.90 peaks/Mb) for the spleen, and 20 (3.12 peaks/Mb) for the cerebellum, and the greatest number of peaks per Mb was on chromosome 7 (4.99 peaks/Mb for the liver, 7.79 peaks/Mb for the spleen, and 4.98 peaks/Mb in the cerebellum) for all three tissues. The average width of broad peak H3K4me1 was greater than for the narrow peaks described above, as expected, at 948 bp for the liver, 2,963 bp for the spleen, and 1,909 bp for the cerebellum. Lastly, the broad mark H3K27me3 had a lower average number of peaks called compared with H3K4me1 (mean of 39,162 in the liver, 29,939 in the spleen, and 26,244 in the cerebellum). The lowest number of H3K27me3 peaks per Mb of chromosome length were on chromosomes 26 (3.04 peaks/Mb), 24 (2.58 peaks/Mb), and 11 (1.84 peaks/Mb) for the liver, spleen, and cerebellum, respectively. The greatest number of peaks was on chromosome 13 (4.86 peaks/Mb) for the liver and chromosome 6 for both the spleen (4.39 peaks/Mb) and cerebellum (4.94 peaks/Mb). The average width of broad H3K27me3 peaks was 440 bp in the liver, 2,143 bp in the spleen, and 653 bp in the cerebellum. Peaks in common across the animals were calculated for all five ChIP-seq experiments and displayed for the liver, spleen, and cerebellum (Supplementary Figure 2). Interestingly, half siblings (F1 and M1, F2 and M2) displayed a greater number of peaks in common with each other.

**FIGURE 2:**
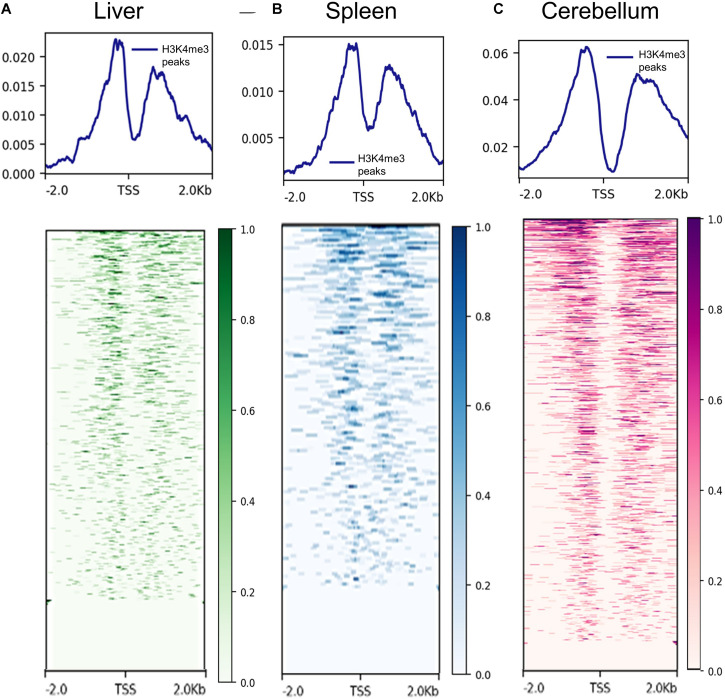
Signal of H3K4me3 ChIP-seq peaks 2 kb upstream and downstream of transcription start sites (TSS) identified by CAGE assays. **(A)** Liver H3K4me3 signal (from F1, M1, and M2 consensus peaks) near TSS annotated in the reference genome, **(B)** spleen H3K4me3 signal (from F2, M1, and M2 consensus peaks) near annotated transcription start sites (TSS), and **(C)** cerebellum H3K4me3 signal (from F1, M1, and M2 consensus peaks) near annotated TSS.

The proximity of H3K4me3 peaks to TSS was investigated by comparing consensus H3K4me3 peaks and CAGE data generated by Salavati et al. (2020). Not surprisingly, H3K4me3 peaks were detected on both sides of the TSS in the liver, spleen, and cerebellum tissues. The signal distributions and heatmaps from 2 kb upstream and downstream of the TSS locations are displayed in Figure 2. In addition, the consensus peaks for H3K4me3, H3K27ac, H3K4me1, H3K27me3, and CTCF were annotated with the *Oar_rambouillet_v1.0* genome annotation file and these classifications are displayed in Supplementary Figures 3–5. The histone modification H3K4me3 had the greatest proportion of peaks annotated as near a TSS when compared with other histone modifications in all three tissues. H3K27ac and H3K4me1 histone modifications displayed intronic annotation most commonly, and H3K27me3 and CTCF displayed mostly intergenic peak annotation.

### Visual Assessment of Sequence Pileup

**FIGURE 3:**
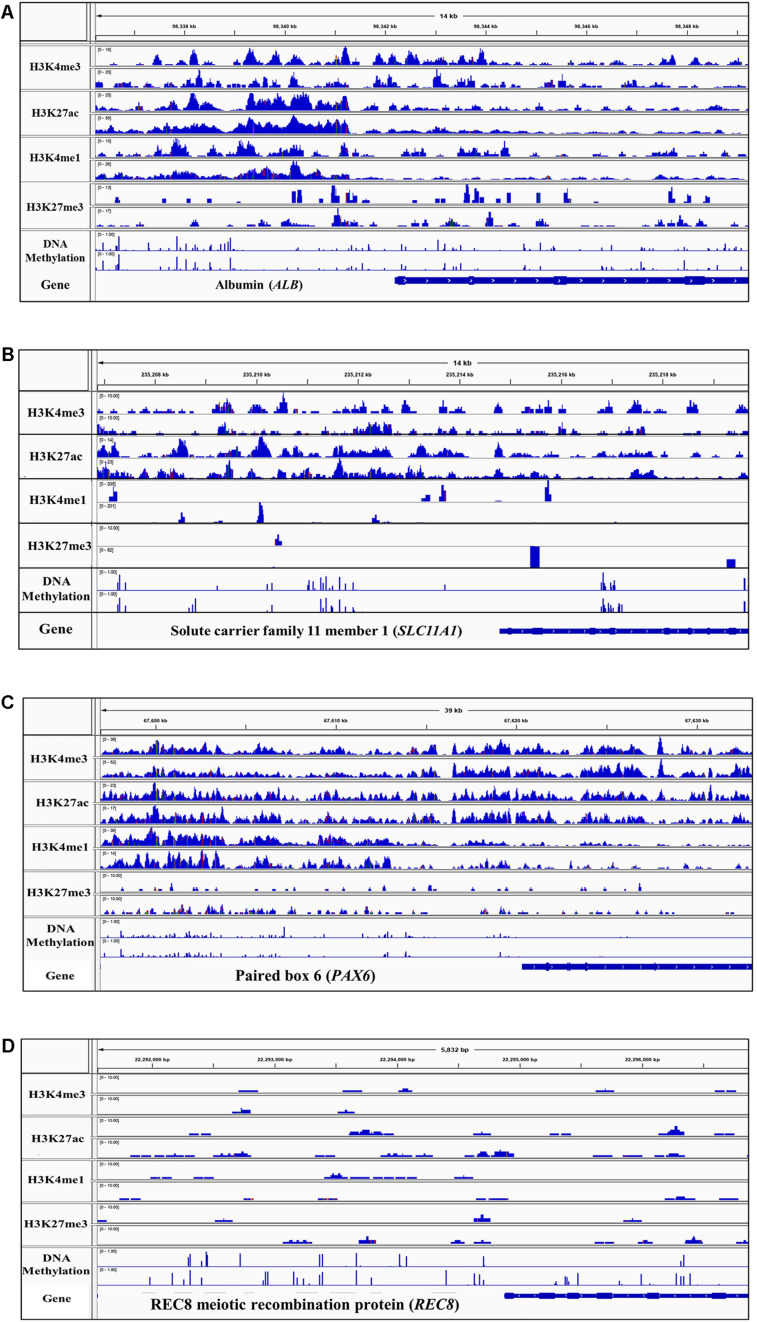
Integrative genomics viewer (IGV) screenshot of sequence pileup normalized with the input control for active and repressive histone marks and DNA methylation in two representative samples (M1 and M2) for **(A)** positive control Albumin (*ALB*) gene in the liver, **(B)** positive control Solute carrier family 11 member 1 (*SLC11A1*) in the spleen, **(C)** positive control Paired box 6 (*PAX6*) in the cerebellum, and **(D)** negative control REC8 gene (*REC8*) in all three tissues.

The peak predictions were directly examined in the IGV (Robinson et al., 2011) for regions known to be active or repressed in the three tissues, to provide an evaluation of the success of the process in properly classifying chromatin states. One example of an expected active region for each liver, spleen, and cerebellum tissue as well as one region expected to be repressed in all tissues is displayed in Figure 3. Albumin (*ALB*), a gene that encodes a plasma protein synthesized in hepatocytes and expected to be active in the liver, has one promoter and two enhancers annotated in humans that are within 2 kb upstream from the start of the gene (Frain et al., 1990; Hayashi et al., 1992; Bernardi et al., 2012; Fagerberg et al., 2014). Sequence pileup for active histone marks in the liver was observed in all four sheep that overlap with approximate locations of regulatory elements of *ALB* in humans, and there were low levels of DNA methylation in these regions (Figure 3A). The region upstream of Solute carrier family 11 member 1 (*SLC11A1*), a gene expected to be active in the spleen and encodes a membrane protein involved with macrophage development, displayed sequence pileup for active marks H3K4me3 and H3K27ac and low levels of DNA methylation directly upstream (Figure 3B) (Hedges et al., 2013). Paired box 6 (*PAX6*) is known to be involved in the development of neural tissues and maturation of granule neurons in the cerebellum and is known to have a promoter and multiple enhancers both upstream and downstream of the gene (Ha et al., 2015; Divya et al., 2016). Furthermore, *PAX6* has greater expression in the cerebellum than other tissues in sheep which is supported by the sequence pileup of active histone marks H3K4me3 and H3K27ac, with some activity of H3K4me1 and little DNA methylation (Figure 3C) (Jiang et al., 2014). In contrast, the REC8 meiotic recombination protein (*REC8*) is a gene that encodes a meiosis-specific protein involved in the synapsis of sister chromatids that is not expected to be active in the liver, spleen, or cerebellum (Xu et al., 2005). This gene location shows no sequence pileup in all four sheep in the liver, spleen, or cerebellum and several methylated regions (Figure 3D).

### Variability in Histone Marks Between Animals

Correlations were calculated for histone marks and for DNA methylation between samples to evaluate interanimal variation in sequence pileup signal for the liver, spleen, and cerebellum (Friedman and Alm, 2012; Siska and Kechris, 2017). Correlations of ChIP-seq data (Spearman) and DNA methylation data (Pearson) averages for all four animals and males only (in parentheses) are provided in Table 1. The narrow mark H3K4me3 was moderately correlated between all four animals in the liver (0.66) and spleen (0.54) and highly correlated in the cerebellum (0.85). In males, H3K4me3 was highly correlated in the liver (0.86), spleen (0.71), and cerebellum (0.88). The narrow mark H3K27ac was highly correlated between samples across all three tissues in the liver (0.89 overall and 0.95 in males), spleen (0.78 overall and 0.84 in males), and cerebellum (0.70 overall and 0.91 in males).

**TABLE 1:** Average correlations of sequencing signal between all four animals.

Tissue	H3K4me3	H3K27ac	H3K4me1	H3K27me3	DNA methylation
Liver	0.66 (0.86)	0.89 (0.95)	0.71 (0.93)	0.58 (0.74)	0.72 (0.76)
Spleen	0.54 (0.71)	0.78 (0.84)	0.47 (0.56)	0.37 (0.44)	0.70 (0.74)
Cerebellum	0.85 (0.88)	0.70 (0.91)	0.82 (0.91)	0.72 (0.83)	0.73 (0.76)

*Spearman correlations were used for ChIP-seq data and Pearson correlations were used for DNA methylation data. Parentheses indicate correlations between the replicates used in the ChromHMM chromatin state analysis.*

The broad mark H3K4me1 also showed high correlation in two tissues, namely the liver (0.71 overall and 0.93 in males) and cerebellum (0.82 overall and 0.91 in males), but the correlation in the spleen was markedly lower (0.47 overall and 0.56 in males), and overall, the correlations between the spleen samples were lower than the liver and cerebellum for all four histone marks. This is evident in H3K27me3 in the spleen (0.37 overall and 0.44 in males) than in the liver (0.58 overall and 0.74 in males) and cerebellum (0.72 overall and 0.83 in males). The correlations of DNA methylation signal between samples ranged from 0.70 to 0.76, with the liver and cerebellum displaying the greatest correlation between the two males (0.76). However, sex differences in correlations were not observed, as each female has a moderate to high correlation with both the other female (0.54–0.84) and both males (0.44–0.92) for each mark within all three tissues.

### Principal Component Analysis of DNA Methylation

**FIGURE 4:**
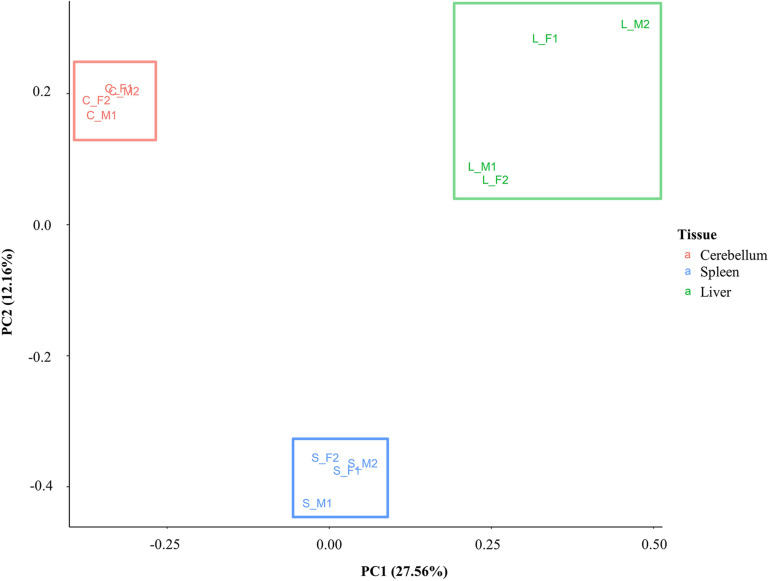
Principal component analysis plot based on CG methylation. Four animals are labeled as F1, F2, M1, and M2. The cerebellum, liver, and spleen samples are labeled as C, L, and S, respectively.

A principal component analysis was performed with the DNA methylation data to investigate similarity and differences between samples and tissues. Eigenvalues were calculated based on the position of CG methylation signal in all animals for all three tissues, and the first two eigenvalues (PC1 and PC2) were plotted (Figure 4). Samples cluster distinctly by tissue type rather than by sex or individual animal. The greatest spread of points within a tissue was observed in the liver. The first eigenvalue (PC1, 27.56%) shows separation of the liver, spleen, and cerebellum. The second eigenvalue (PC2, 12.16%) shows another dimension of separation of the cerebellum and liver from the spleen.

### Methylation Level at CG and Non-CG Sites

**FIGURE 5:**
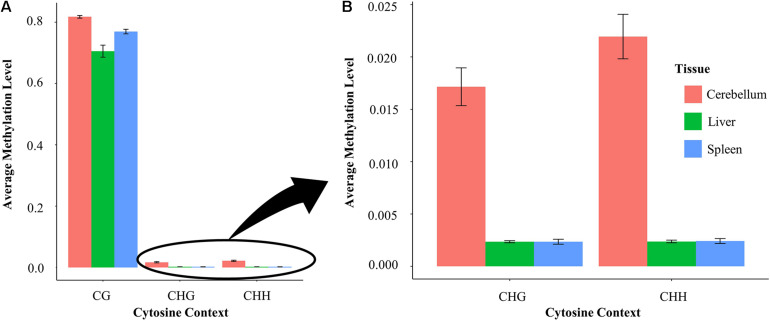
**(A)** Methylation level at CG compared with non-CG sites in the liver, spleen, and cerebellum and **(B)** methylation level at non-CG (CHG and CHH) sites in each tissue enlarged.

Average methylation levels were calculated and compared in each of the three tissues in both the CG and non-CG sites (Figure 5A). Non-CG sites are defined as CHG and CHH where H is either A/T/C. CG sites have an average methylation level ranging between 70 and 81% across different tissues. Specifically, cerebellum samples have an average methylation level of 81.4%, whereas liver and spleen samples have an average methylation level of 70.3 and 76.9%, respectively. The average methylation level of cytosines at non-CG contexts (CHG and CHH) is nine-fold higher in the cerebellum (1.7–2.1%) than in spleen and liver samples (0.2%) (Figure 5B).

### Chromatin State Assignment and Correlation With Methylation Status

The relative positions of the combination of specific histone marks provide a more complete definition of the overall regulatory chromatin state than individual peak calling. Regulatory elements were defined for two animals (M1 and M2) using a hidden Markov model employed by ChromHMM which assigns 200 bp bins across the genome to a given number of chromatin states based on the combined histone modification signal profiles (Ernst and Kellis, 2010, 2017). The genome was categorized into two through 20 chromatin states using ChromHMM. The optimal number of states was determined to be nine, as it was the lowest number of states that had greater than 0.95 correlation of all samples to 20 states, which captures the complexity of the data with fewer states (see Supplementary Figure 2) (Gorkin et al., 2017, 2020). These nine chromatin states are categorized as follows: promoter, active enhancer, poised enhancer, repressed enhancer, CTCF, and three or four states of quiescent/low signal. The consensus of chromatin states assigned to both M1 and M2 was used for further analyses.

**FIGURE 6:**
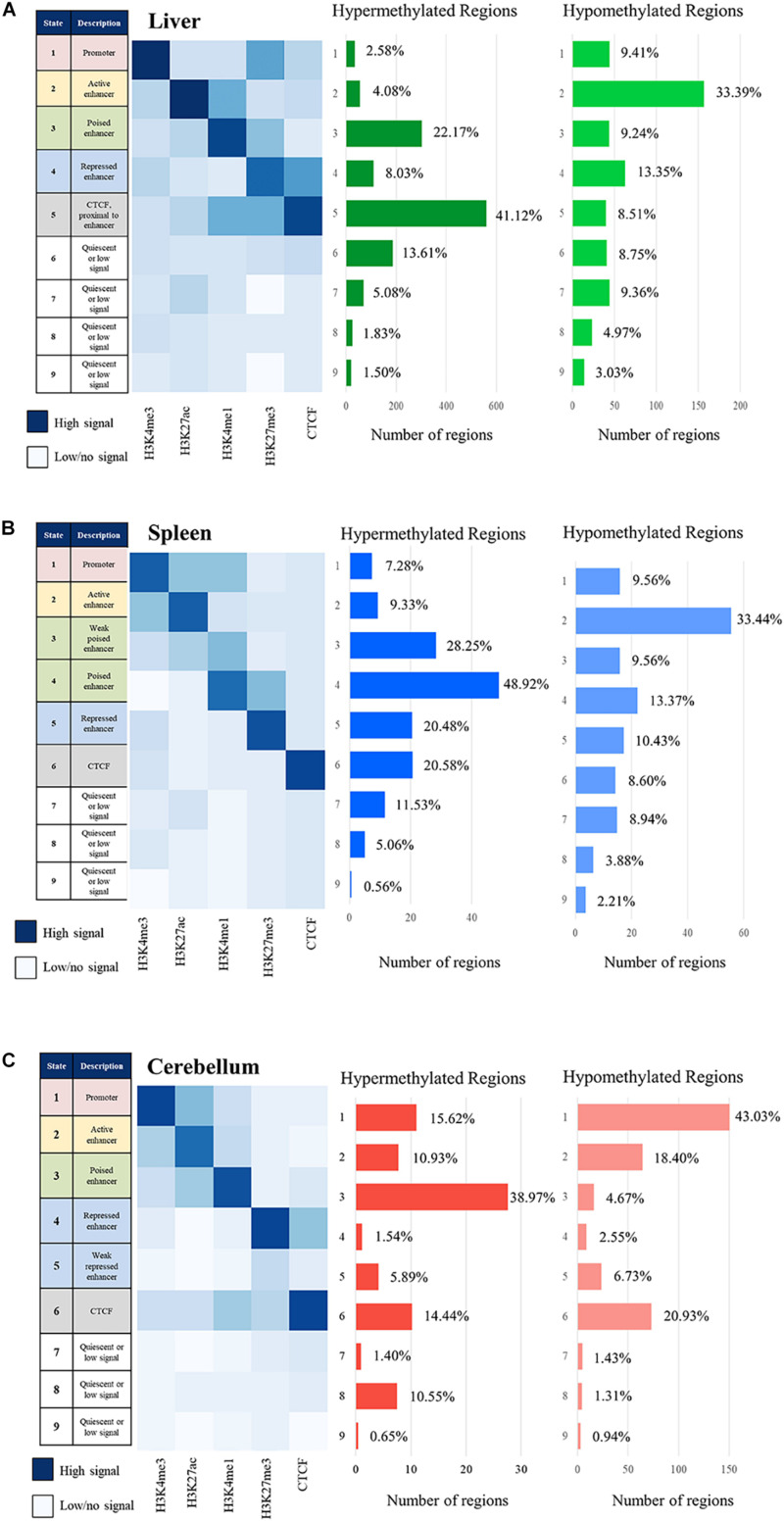
Chromatin state description and ChromHMM heatmap with histone mark signal overlap consensus from M1 and M2 compared with the number of hypermethylated regions and hypomethylated region consensus per Mb for M1 and M2 for the **(A)** liver, **(B)** spleen, and **(C)** cerebellum.

The signal of all the histone marks and the nine chromatin states for each tissue is displayed as heatmaps in Figure 6. Regions with primarily H3K4me3 signal often overlapping with H3K27ac are considered promoters, regions with strong H3K27ac signal are considered active enhancers, regions with H3K4me1 often paired with weak H3K27me3 signal are considered poised enhancers, and regions with strong H3K27me3 signal are considered repressed enhancers (Wang et al., 2008; Creyghton et al., 2010; Core et al., 2014; Carelli et al., 2018). All four of these categories of regulatory elements were observed and displayed in the heatmaps, with the addition of a weak poised enhancer state in the spleen and weak repressed enhancer state in the cerebellum which both displayed lower but still distinguishable signal. In addition, regions with CTCF signal which overlap with other marks including H3K4me1 and H3K27me3 were observed in the liver and cerebellum. Lastly, quiescent/low states had very little signal in any of the five marks.

The correlation of DNA methylation status with predicted chromatin state was examined by estimating the number of hyper- and hypomethylated regions per Mb within the boundaries of the regulatory elements in the nine defined chromatin states. The greatest number of hypomethylated regions was observed in active enhancer regions in the liver and spleen and in active promoter regions in the cerebellum, as expected if our process was correctly identifying regulatory elements and classifying them as actively transcribed genes. The greatest number of hypermethylated regions was observed in poised enhancers and CTCF in the liver, weak poised and poised enhancer regions in the spleen, and poised enhancer regions in the cerebellum, also consistent with the process correctly classifying regulatory elements.

### Distribution of Chromatin States in the Genome and Proximity to TSS

**FIGURE 7:**
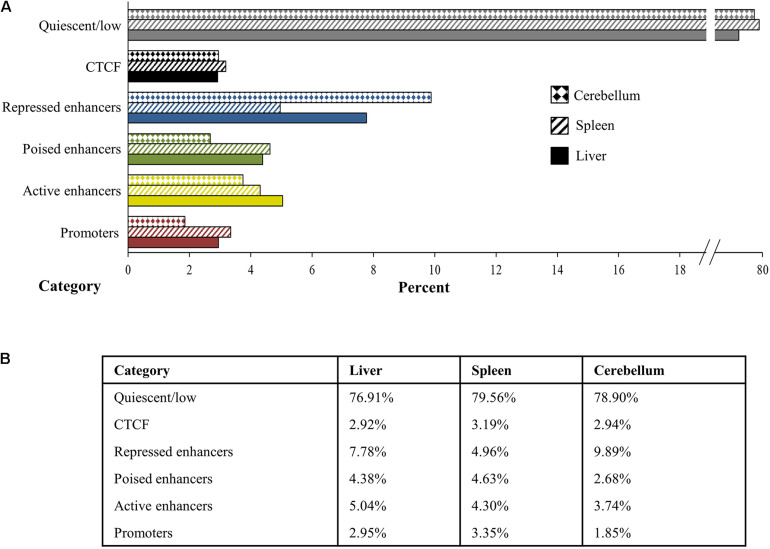
Percent of the genome in the liver, spleen, and cerebellum (from M1 and M2) assigned to each category of quiescent/low (gray), CTCF (black), repressed enhancer (blue), poised enhancer (green), active enhancer (gold), and promoter (red) depicted visually in panel **(A)** the bar graph and numerically in panel **(B)** the table.

The chromosomal segments spanned by regulatory elements, as defined by the histone mark peaks, were combined and summarized to estimate the overall extent and percent of the genome representing regulatory elements and their chromatin state among the three tissues examined. Chromatin states from the ChromHMM analyses were categorized and combined into promoter, active enhancer, poised enhancer including weak poised enhancers, repressed enhancer including weak repressed enhancers, and quiescent or low signal categories and averaged for each tissue (Figure 7). Promoters comprise 2.95% of the genome in the liver, 3.35% in the spleen, and 1.85% in the cerebellum, and active enhancers occupy 5.04% of the genome in the liver, 4.30% in the spleen, and 3.74% in the cerebellum. In addition, 4.38% of the genome in the liver, 4.63% in the spleen, and 2.68% in the cerebellum are categorized as poised enhancers, while 7.78% of the genome in the liver, 4.96% in the spleen, and 9.89% in the cerebellum are considered repressed enhancers. The percent of the genome that had primarily CTCF signal was 2.92% in the liver, 3.19% in the spleen, and 2.94% in the cerebellum. Cumulatively, states considered as enriched with histone mark and CTCF signal intensity by ChromHMM, which includes the promoter, enhancer, and CTCF functional elements, comprise approximately 23.08% of the genome in the liver, 20.44% in the spleen, and 21.10% in the cerebellum. Not surprisingly, the largest percent of the genome, 76.91% in the liver, 79.56% in the spleen, and 78.90% in the cerebellum, was categorized as quiescent or low signal.

The locations of assigned promoter chromatin states were compared with TSS generated from CAGE data for the liver, spleen, and cerebellum. Both the signal distribution and heatmap plots display a strong signal before and after the TSS in all three tissues (Supplementary Figure 7). This signal is similar to the distribution of the H3K4me3 peak signal before and after TSS, which is not surprising as the ChromHMM model assigns promoter states based on the presence of H3K4me3 signal. It is worth noting that the CAGE data used in this study were generated from the reference genome animal, a Rambouillet, which is different from the crossbred animals used in this study and may explain some of the signal noise.

### Similarities and Differences of Chromatin States Between Tissues

**FIGURE 8:**
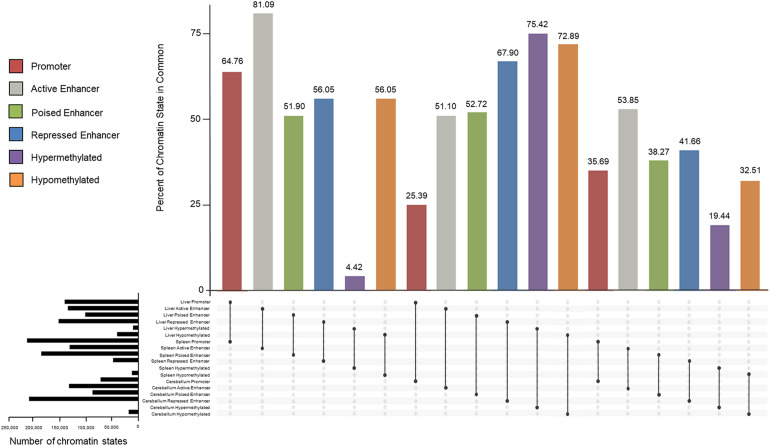
Percent of overlapping promoter (red), active enhancer (gray), poised enhancer (green), and repressed enhancer (blue) chromatin state categories and hypermethylated (purple) and hypomethylated (orange) regions between the liver, spleen, and cerebellum tissues of the consensus categories from M1 and M2. The total number of chromatin states for each tissue is displayed in black horizontal bars.

Similarities and differences of promoters, enhancers, and methylated regions within and between tissues were examined and percentages of overlap are displayed in Figure 8. Active promoters were 64.76% similar between the liver and spleen, 25.39% between the liver and cerebellum, and 35.69% between the spleen and cerebellum. The liver had 81.09 and 51.10% of active enhancers in common with the spleen and cerebellum, respectively. The spleen and cerebellum had 53.85% similarity of active enhancers. Poised enhancers were shared 51.90% between the liver and spleen, 52.72% between the liver and cerebellum, and 38.27% between the spleen and cerebellum. The percent of repressed enhancers that overlapped between the liver and spleen was 56.05%. The liver and cerebellum repressed enhancers overlapped 67.90%, and the spleen and cerebellum repressed enhancers overlapped 41.66%. Hypermethylated genomic locations overlapped 4.42% and hypomethylated regions overlapped 56.05% between the liver and spleen. The liver and cerebellum displayed more similar hypermethylated and hypomethylated regions, 75.42 and 72.89%, respectively, than the spleen and cerebellum, 19.44 and 32.51%, respectively.

### CTCF-Binding Motifs

The insulator CTCF is often present at the boundaries of topologically associated domains (TADs), compartments of chromatin interactions, across the genome (Beagan and Phillips-Cremins, 2020). The location of significant (*P* < 0.00001) CTCF-binding motifs both known from previous research and *de novo* was identified across the genome in the liver, spleen, and cerebellum (Heinz et al., 2010). Of these, 13 were present in at least three animals (Table 2). Three motifs, MYB3R4, MYB3R1, and Pdx1, were significantly enriched in the liver, spleen, and cerebellum tissues. The liver and spleen exhibited the most significantly enriched CTCF motifs in common (TAGL, Six2, RRTF1, Sox6, SVP, and TGA2). One motif, ZBTB19, was enriched in the spleen and cerebellum. The cerebellum had three enriched motifs (Elk4, Pho2, and BZR1) not present in the liver or spleen. In addition, *de novo* motifs were identified in all three tissues. The top three most significant *de novo* motifs per sample in the liver, spleen, and cerebellum are reported in Tables 3–5, respectively. Of the total number of *de novo* motifs, 16, 13, and 21 were identified as unique to the liver, spleen, and cerebellum, respectively. Sixteen *de novo* motifs were identified in both the liver and spleen, while the cerebellum had only three *de novo* motifs in common with the other tissues.

**TABLE 2:**
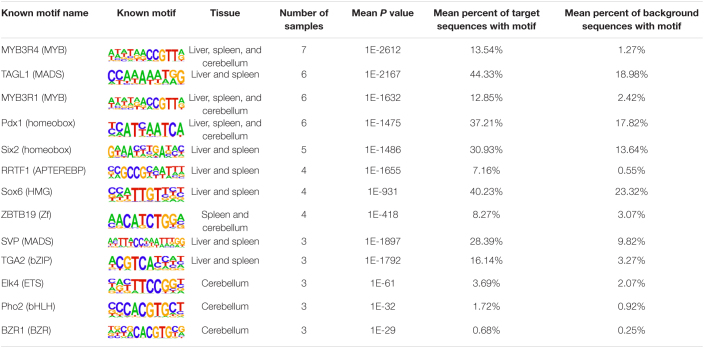
Known CTCF motifs present in the top 10 most significant motifs across multiple samples.

**TABLE 3:**
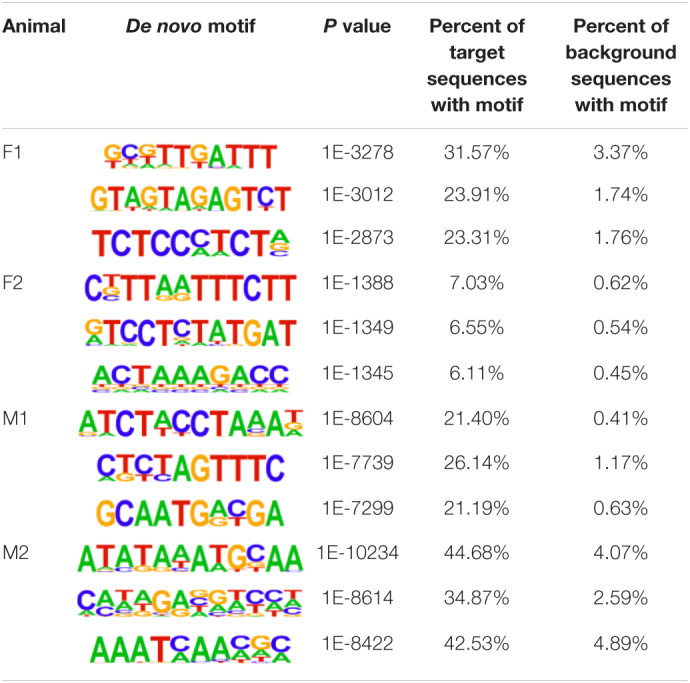
Top three *de novo* CTCF motifs present in each sample in the liver.

**TABLE 4:**
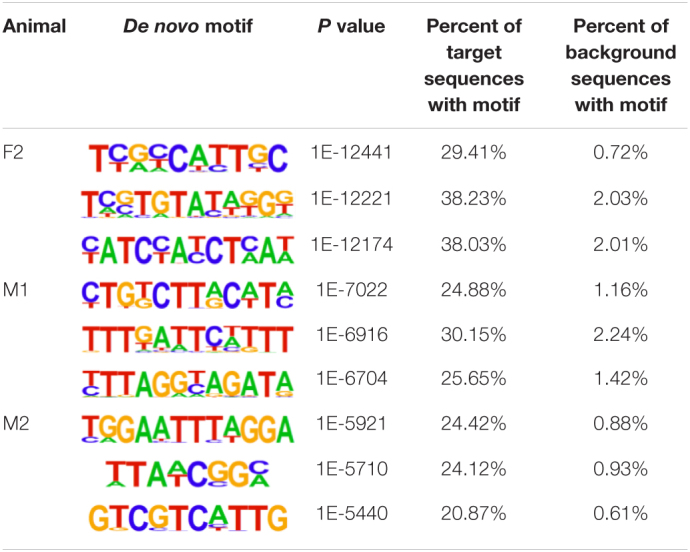
Top three *de novo* CTCF motifs present in each sample in the spleen.

**TABLE 5:**
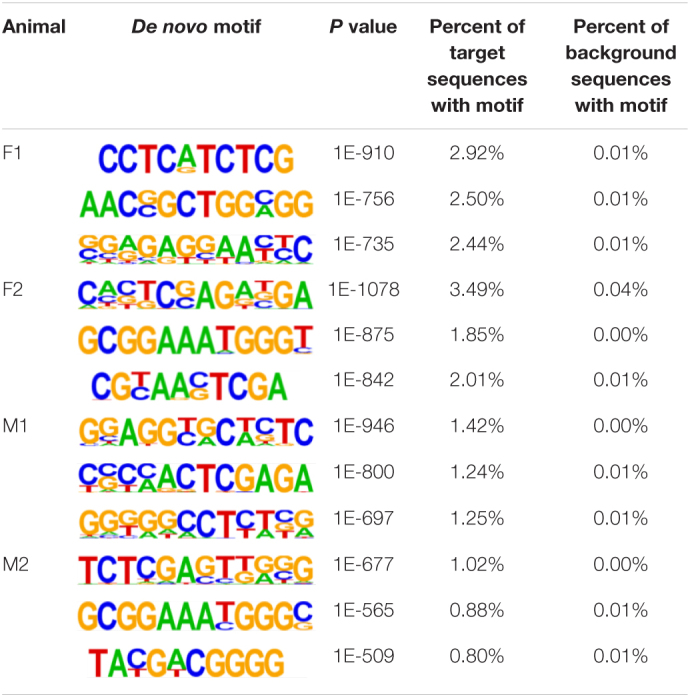
Top three *de novo* CTCF motifs present in each sample in the cerebellum.

## Discussion

The goal of this study was to characterize regulatory elements in ovine liver, spleen, and cerebellum using ChIP-seq and WGBS. The three selected tissues, the liver, spleen, and cerebellum, each represent a different developmental origin and are important to metabolism, immune response, and motor control, respectively. We have demonstrated the successful application of the micrococcal nuclease ChIP protocol across these tissues and the bioinformatic pipeline for the analysis of ChIP-seq in sheep. Furthermore, this study has incorporated the value of coupled histone modification and DNA methylation data toward a better understanding of regulatory regions in the sheep genome.

Micrococcal nuclease was used to shear the chromatin because it provided a consistent and reproducible shearing across samples and tissue types. A limitation of the micrococcal nuclease may be increased likelihood of the appearance of duplicated reads due to similarity of cut sites in the chromatin; however, several studies have not found substantial bias when duplicates were removed (Allan et al., 2012; David et al., 2017; Gutiérrez et al., 2017; Chereji et al., 2019). Furthermore, shearing with micrococcal nuclease to approximately 1–2 nucleosome lengths may contribute to slightly different characteristics, including width, of peaks called from these experiments.

Sequence read pileups were examined in IGV near genes known to be active and inactive in humans and expected to be conserved across species. This provided a means of examining genes with known promoters and expression patterns as positive and negative controls for both ChIP-seq experiments and WGBS and provided insight into the potential similarity of regulatory elements across species. Several genes known to be active across different mammalian species in the liver, spleen, and cerebellum showed a sequence pileup of active histone marks which likely indicated the presence of active regulatory elements. Inversely, genes known to be active during meiotic processes and quiescent during adult stages in several mammalian species showed no sequence pileup of histone marks and presence of DNA methylation, which likely indicates inactivity of regulatory elements.

Consistency of regulatory element identification by ChIP-seq and DNA methylation for each tissue between the four individual animals was evaluated by calculating Spearman and Pearson correlations, respectively. Correlations between samples for both ChIP-seq and DNA methylation were within the ranges previously reported with sequence data (Peng et al., 2010; Siska and Kechris, 2017). Furthermore, correlations between ChIP-seq biological replicates have been reported as low as 0.3–0.4, with technical replicates reported as high as 0.9 (Friedman and Alm, 2012; Siska and Kechris, 2017). The results for these sheep tissues therefore achieve equivalent or improved results compared with previously reported pipelines for regulatory element identification and characterization and demonstrate a tissue-specific moderate variation across biological replicates. The spleen displayed the highest variation between biological replicates, with correlations between 0.44 and 0.84 among histone marks, although DNA methylation was consistent across replicates including the spleen. Given that splenic tissue is an acutely responsive immunological tissue, perhaps it is not surprising that we observed greater variation in the biological replicates.

The CG methylation signal for all four samples clustered distinctly by tissue in a principal component analysis, indicating clear differences in DNA methylation between tissues (Figure 3). This finding is supported by others that have shown that the greatest differences in methylation occur between tissue types rather than between individuals (Pai et al., 2011; Zhang et al., 2013) and consistent with the requirement for a particular set of genes to be active and therefore demethylated depending on tissue function. Cerebellum samples demonstrated a higher level of both CG and non-CG methylation compared to the liver and spleen. Brain tissues are known to differ from other tissues in methylation patterns in other species, and furthermore, the cerebellum has been shown to be different than other brain tissues (Gibbs et al., 2010; Cantrell et al., 2019).

The enrichment of individual histone marks was examined by identifying peaks in each sample. The number of peaks identified in these sheep liver, spleen, and cerebellum samples was consistent with other studies in sheep adipose, cattle liver, cattle muscle, cattle rumen epithelium, human liver, and mouse liver (Supplementary Table 3) (Villar et al., 2015; Zhao et al., 2015; Naval-Sanchez et al., 2018; Fang et al., 2019). Many chromosomes had differences in peak numbers normalized by chromosome length between tissues, indicating potential tissue specificity of some peaks. Narrow marks H3K3me3, H3K27ac, and CTCF had a shorter average width than broad marks H3K4me1 and H3K27me3, which may be influenced by the program and statistical model used to call peaks as well as by the shearing method (Zhang et al., 2008, 2009). Because micrococcal nuclease was used for shearing, the length of the narrow peaks more closely resembles the size of a single nucleosome.

Trimethylation of histone 3 lysine 4 peaks were enriched annotated TSS in all three tissues. The peaks and heatmap signature signals are similar to several other ChIP-seq experiments in human PBMCs and CD14+ cells, as well as mouse liver (Schones et al., 2008; Quinodoz et al., 2014; Uchiyama et al., 2018). Peaks from all histone modifications and CTCF were also annotated with regions defined in the *Oar_rambouillet_v1.0* genome. In the liver, spleen, and cerebellum, the most TSS were identified near (within 2 kb of distance on either side) to H3K4me3 peaks, which is not surprising. Many H3K27ac and H3K4me1 peaks, which indicate the presence of active or poised enhancers, were located in intronic regions. Repressed enhancers marked by H3K27me3 were located mostly in intergenic regions, along with CTCF, which may be indicative of insulated TAD boundaries not in close proximity of genes. Further work with additional animals in combination with RNA expression and TSS analyses is needed to examine regulatory element activity outside of previously annotated regions of the sheep genome.

The genomic segments identified by histone mark peaks were evaluated for overlap between marks and CTCF binding. This broader view of the regulatory landscape lends a better understanding of gene regulation at each location than individual marks (Park, 2018). Active promoters have been shown to exhibit greater enrichment of H3K4me3 than other histone marks in addition to the often present H3K27ac (Wang et al., 2008; Creyghton et al., 2010; Carelli et al., 2018). However, if lysine 4 is monomethylated (H3K4me1), this indicates the presence of a poised enhancer, in which enrichment of lysine 27 can be acetylated or trimethylated depending on the state and activity of the enhancer (Heintzman et al., 2007; Wang et al., 2008; Creyghton et al., 2010; Carelli et al., 2018). Low H3K4me3 coincident with high H3K27ac signal has been reported to be common at enhancers near genes undergoing highly active transcription (Core et al., 2014; Carelli et al., 2018). Repressed enhancers are generally characterized by H3K27me3 signal (Carelli et al., 2018). However, H3K27me3 has also been shown to be enriched near the promoter or gene body in genes being expressed at a relatively low rate (Young et al., 2011; Flensburg et al., 2014). The chromatin states characterized in this study are similar to what others have previously described in cattle (Fang et al., 2019). Furthermore, the weak poised enhancer category detected in the spleen and the weak repressed enhancer category detected in the cerebellum demonstrate that different tissues may have varying chromatin states, which supports the importance of characterizing chromatin states across tissues within a species.

Hypermethylated and hypomethylated regions of the sheep genome were defined across liver, spleen, and cerebellum tissues. The number of hypermethylated and hypomethylated regions per Mb in each of the nine chromatin states was quantified. The data presented in this study demonstrate an enrichment of hypermethylated regions in chromatin states with prominent H3K4me1 (primarily poised enhancers) and hypomethylated regions in active enhancers and promoters enriched with H3K27ac and H3K4me3. These results agree with previous research in humans and mice which indicate that active enhancer activity is inversely correlated with DNA methylation (Aran and Hellman, 2013; Barwick et al., 2016; Bell and Vertino, 2017). Interestingly, the presence of H3K4me1 was found to be positively correlated with DNA methylation, specifically intermediate methylation (25–75%), in mice (Zhang et al., 2009; Teng and Tan, 2012; Sharifi-Zarchi et al., 2017). Furthermore, enhancers enriched with H3K27ac and promoters enriched with H3K4me3 had less DNA methylation than other regions (Sharifi-Zarchi et al., 2017).

Approximately 20% of the sheep genome was assigned to a chromatin state category including promoters; active, poised, and repressed enhancers; and CTCF in the liver, spleen, and cerebellum. In cattle, a previous study similarly assigned approximately 30% of the genome to either a chromatin state or areas with open chromatin in rumen epithelium (Fang et al., 2019). The locations of many regulatory elements were similar between the liver and spleen in this study; however, a greater difference was observed in active enhancers and promoters between the cerebellum compared with the liver and spleen. Since distinct differences in gene expression and regulation have been observed between the cerebellum and other tissues in sheep, this difference is not surprising (Jiang et al., 2014).

The CCCTC-binding factor (CTCF) along with cohesins was shown to be present at the boundaries of TADs in humans and mice (Dixon et al., 2012; Phillips-Cremins et al., 2013; Rao et al., 2014; Vietri Rudan et al., 2015; Szabo et al., 2019). Depending on the cell type, 75–95% of TAD boundaries defined by Hi-C chromatin capture have shown CTCF signal in mice (Bonev et al., 2017; Szabo et al., 2019). The chromatin states in this study that display primarily CTCF could be representative of these domain boundaries; however, Hi-C data are required to confirm which will be possible for the data produced in the FAANG study of the reference ewe, where Hi-C data are also available. In addition to helping define TAD boundaries, CTCF has also been identified near enhancers and promoters within TADs in humans and mice, which then form smaller loop domains with cohesins and the protein YY1 (Phillips-Cremins et al., 2013; Weintraub et al., 2017; Szabo et al., 2019). The chromatin state analysis may be detecting some of these within-TAD loop interactions, with overlap between CTCF and H3K27me3 as well as H3K4me1 signal shown in the chromatin state heatmaps in the liver and cerebellum. Signal from CTCF, H3K27me3, and H3K4me1 marks within one chromatin state was also observed in another study in cattle rumen epithelial tissue and Madin–Darby bovine kidney epithelial cells (Fang et al., 2019).

Motif analysis of CTCF resulted in both known and *de novo* motifs identified in more than one tissue. A large number of CTCF-binding motifs are similar in sequence across mammalian species including cattle (Filippova et al., 1996; Schmidt et al., 2012; Wang et al., 2018). Wang and associates identified putative CTCF-binding motifs in the bovine genome with 82 CTCF motif profiles with similar sequence in human, mouse, dog, and macaque (Schmidt et al., 2012; Wang et al., 2018). In this study, significant motifs identified in ovine liver, spleen, and cerebellum were also identified in human, mouse, fly (*Drosophila melanogaster*), and yeast (*Saccharomyces cerevisiae*) within the HOMER motif database (Heinz et al., 2010).

This experiment examines regulatory elements in multiple sheep tissues and individuals with ChIP-seq and WGBS methylation assays. These data provide putative categories of biological functions for regulatory DNA and will facilitate the identification of epigenetic variation that controls phenotypic traits in sheep. Epigenetic annotation is especially important for revealing the biology behind interesting complex traits since genetic variation does not always reveal the entire story. Epigenetic variation may play a larger role in traits uniquely expressed in a specific tissue or recently evolved rare traits. Identification of causal regulatory variants will allow more rapid genetic improvement for health and production traits in the meat, milk, and wool industries across sheep populations. Causal variants have the highest utility across breeds and allow more efficient assimilation of genetic markers into marker-assisted selection and genomic selection algorithms. The protocols and analysis pipeline optimized here for validation and the eventual annotation of DNA regulatory elements are valuable resources for the Ovine FAANG Project Consortium and the International Sheep Genomics Consortium.

## Members of the Ovine FAANG Project Consortium (listed by institution)

Brenda Murdoch (University of Idaho)
Kimberly Davenport (University of Idaho)
Stephen White (USDA, ARS, ADRU, Washington State University)
Michelle Mousel (USDA, ARS, ADRU, Washington State University)
Alisha Massa (Washington State University)
Kim Worley (Baylor College of Medicine)
Alan Archibald (The Roslin Institute, University of Edinburgh)
Emily Clark (The Roslin Institute, University of Edinburgh)
Brian Dalrymple (University of Western Australia)
James Kijas (CSIRO)
Shannon Clarke (AgResearch)
Rudiger Brauning (AgResearch)
Timothy Smith (USDA, ARS, MARC)
Tracey Hadfield (Utah State University)
Noelle Cockett (Utah State University)

## Data Availability Statement

The datasets presented in this study can be found in online repositories. The names of the repository/repositories and accession number(s) can be found below: NCBI BioProject, accession no: PRJEB41457.

## Ethics Statement

Sheep were housed and cared for under Animal Subject Approval Form (ASAF) 04618 titled “Maintenance of MCF and OPPV free sheep flocks” approved by the Washington State University Institutional Animal Care and Use Committee (IACUC) and euthanized for tissue collection under ASAF 6003.

## Author Contributions

MM, MH, SW, SM, TS, and BM designed the study. KD, AM, MM, MH, and BM collected the samples. BM, KD, MM, SW, AM, SM, and SB facilitated the ChIP-Seq and methylation experiments, data analyses, and drafted the manuscript. TS facilitated ChIP-seq library preparation and sequencing. KD, AM, SB, SM, MM, MH, SW, TS, and BM discussed and interpreted results. SM, MM, SW, NC, TS, and BM acquired funding. All authors contributed to the article and approved the final version.

## Conflict of Interest

The authors declare that the research was conducted in the absence of any commercial or financial relationships that could be construed as a potential conflict of interest.
