# Supplementary figures and images for: Characterizing Genetic Regulatory Elements in Ovine Tissues
Source: Front Genet. 2021 May 20;12:628849. doi: 10.3389/fgene.2021.628849 (PMC8173140; doi:10.3389/fgene.2021.628849)

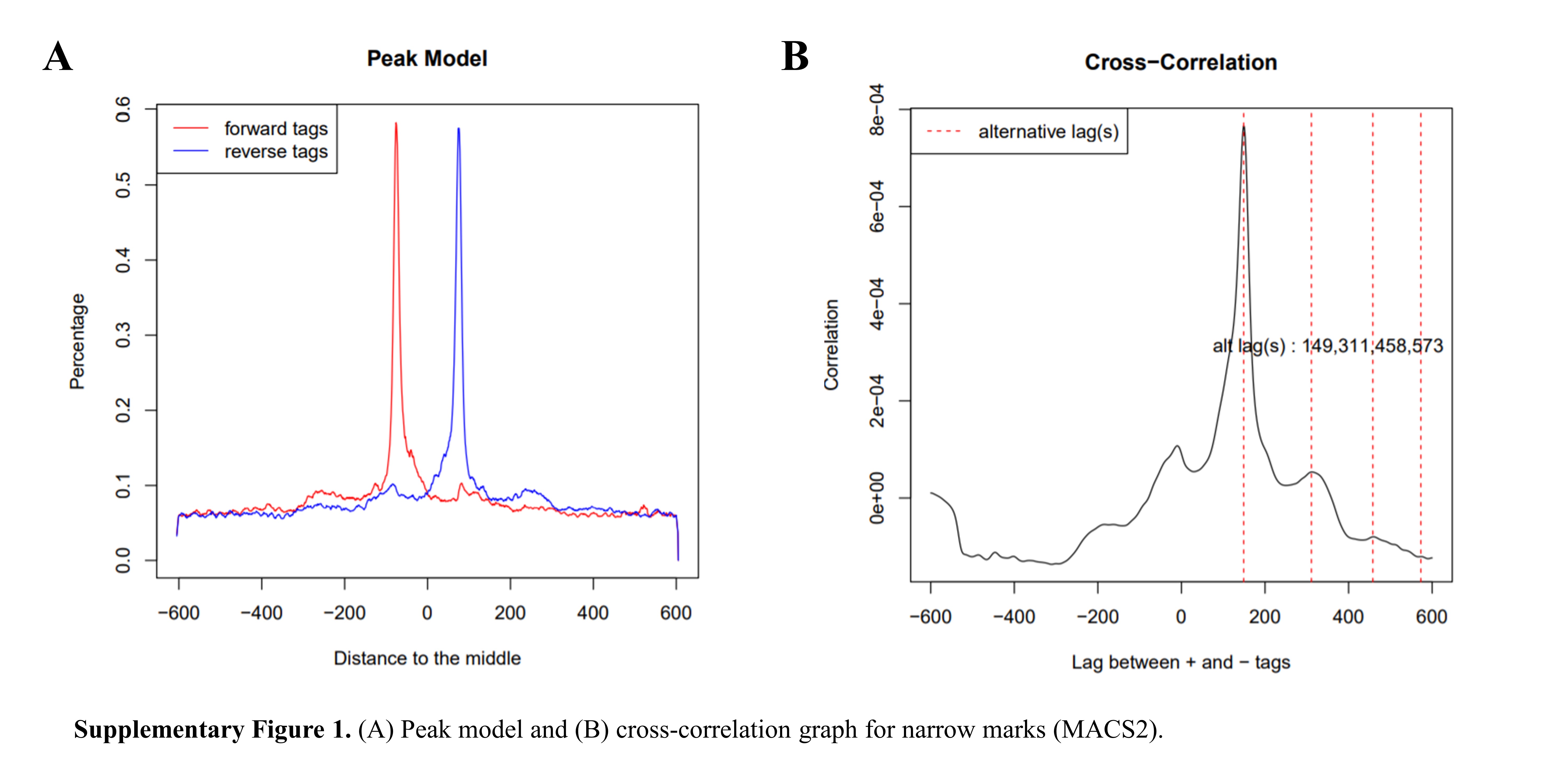

Supplement: Supplementary file 1 [file Image_1.JPEG]

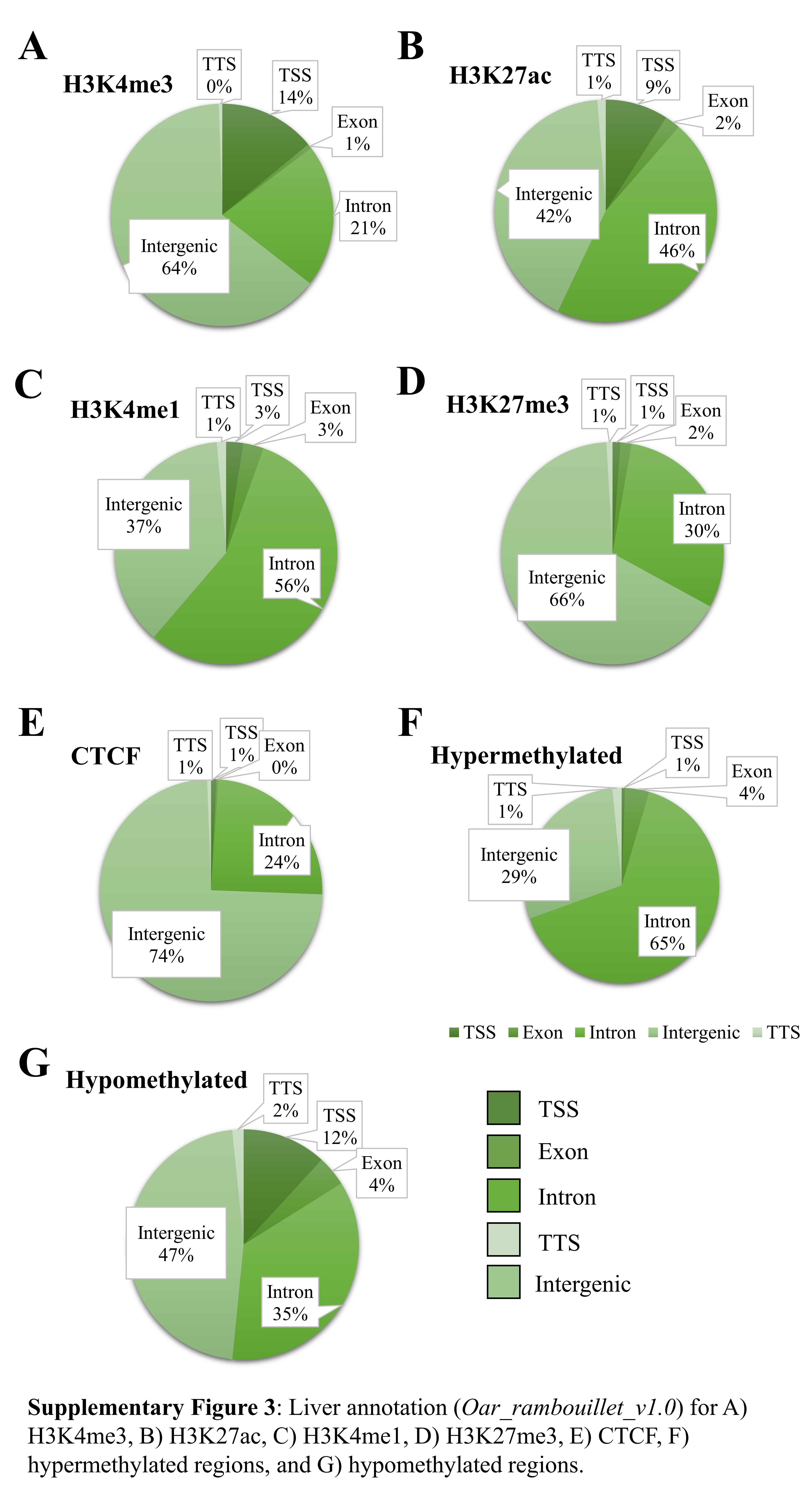

Supplement: Supplementary file 3 [file Image_3.JPEG]

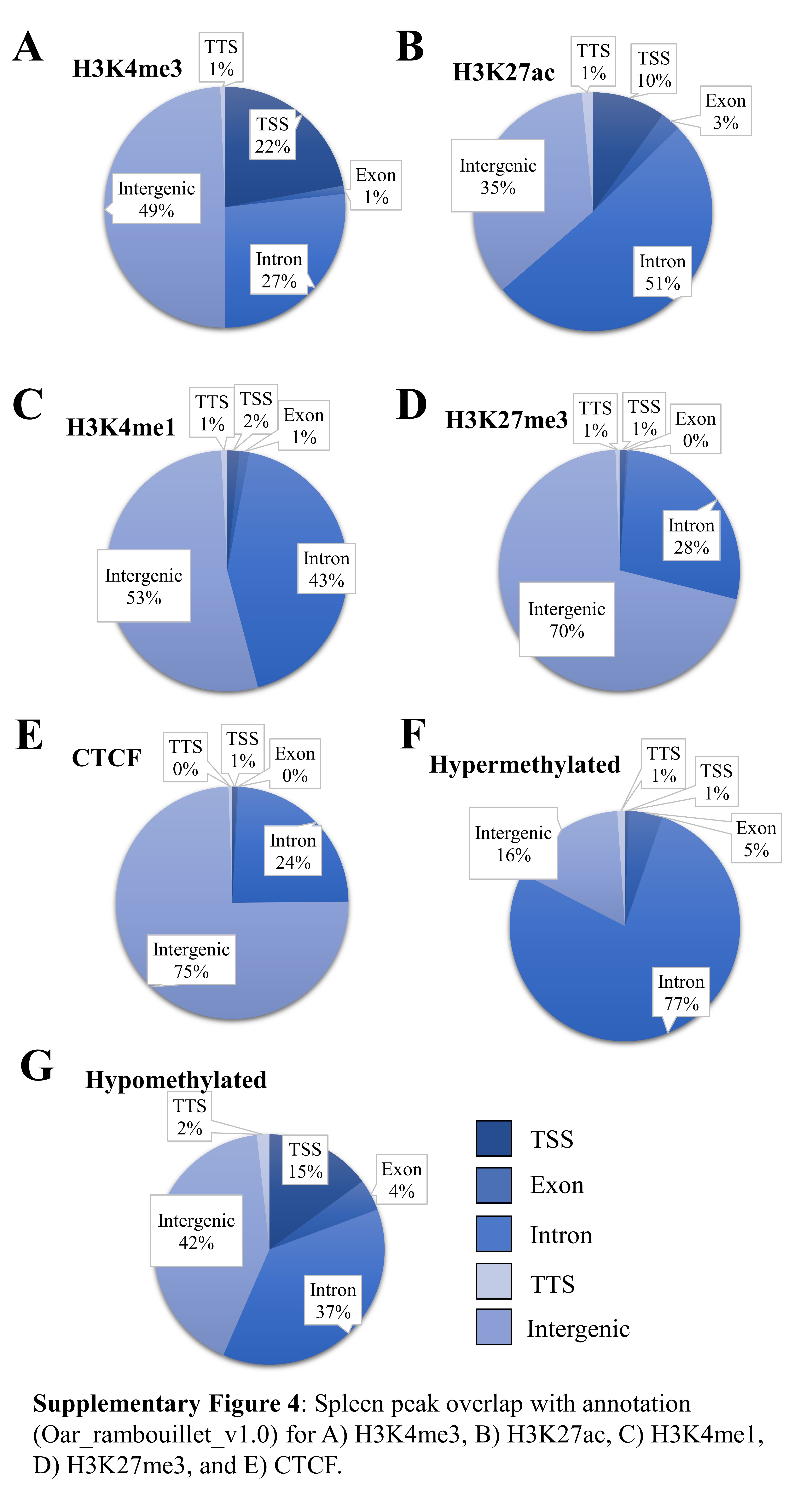

Supplement: Supplementary file 4 [file Image_4.JPEG]

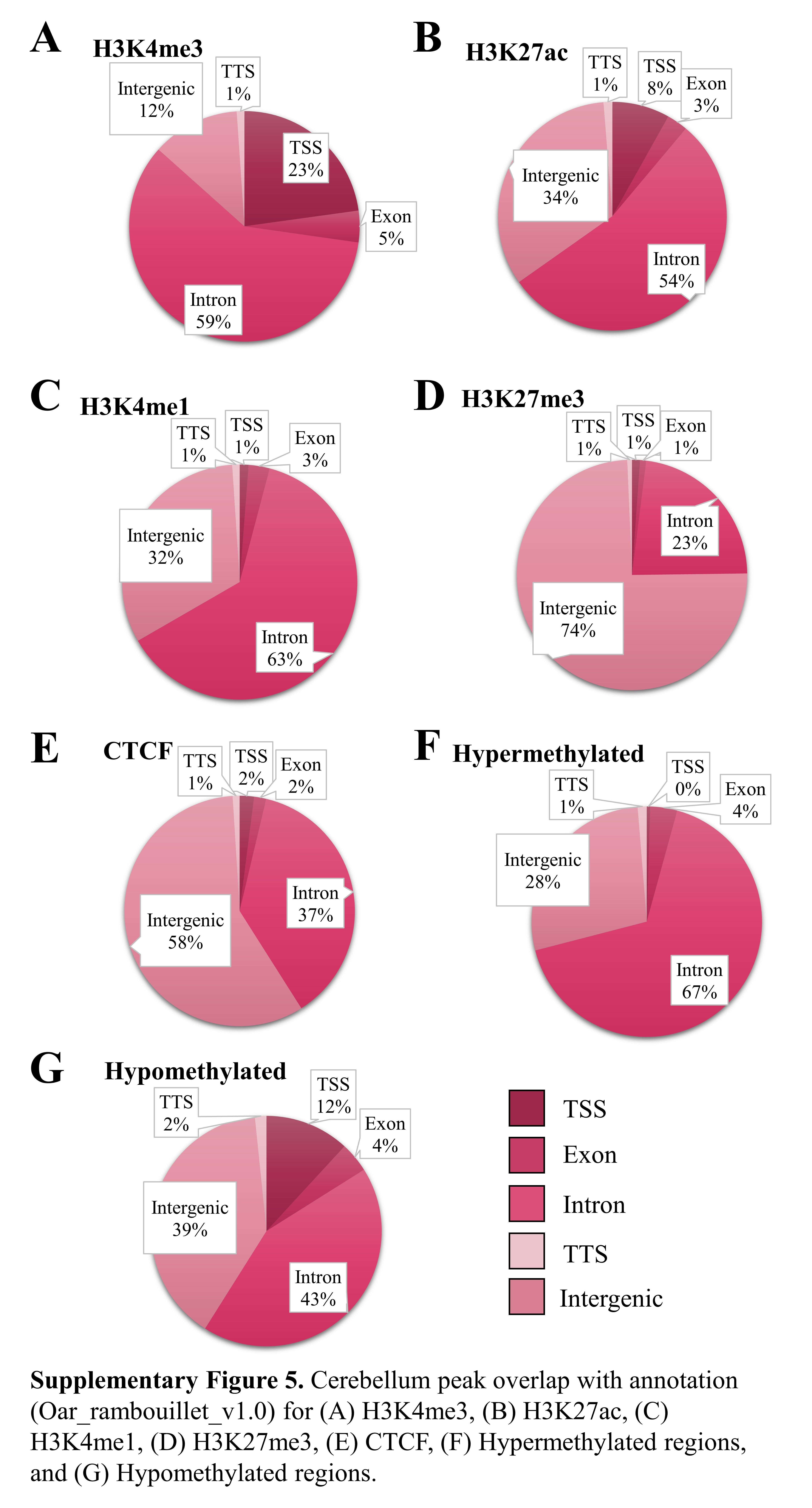

Supplement: Supplementary file 5 [file Image_5.JPEG]

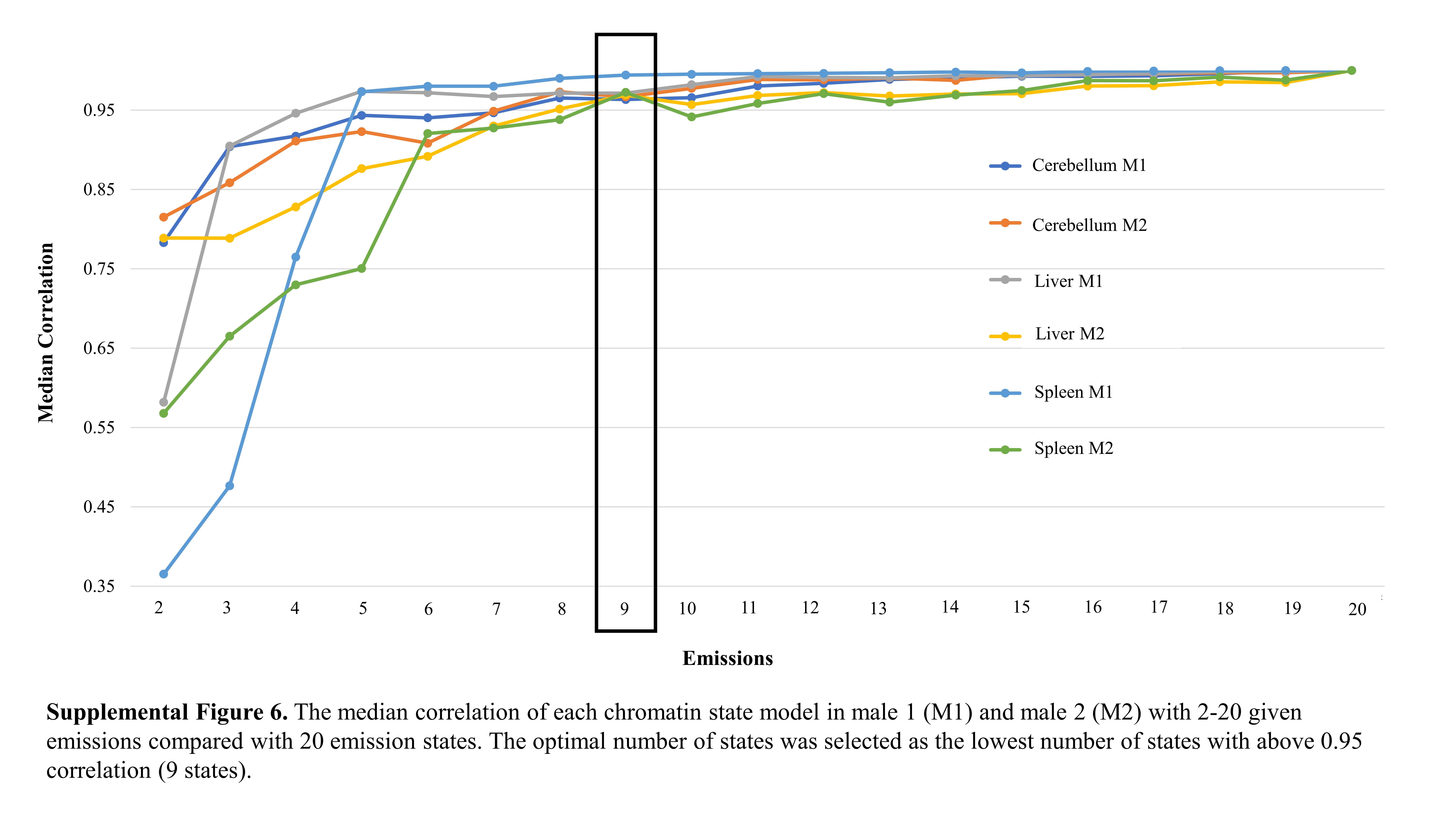

Supplement: Supplementary file 6 [file Image_6.JPEG]

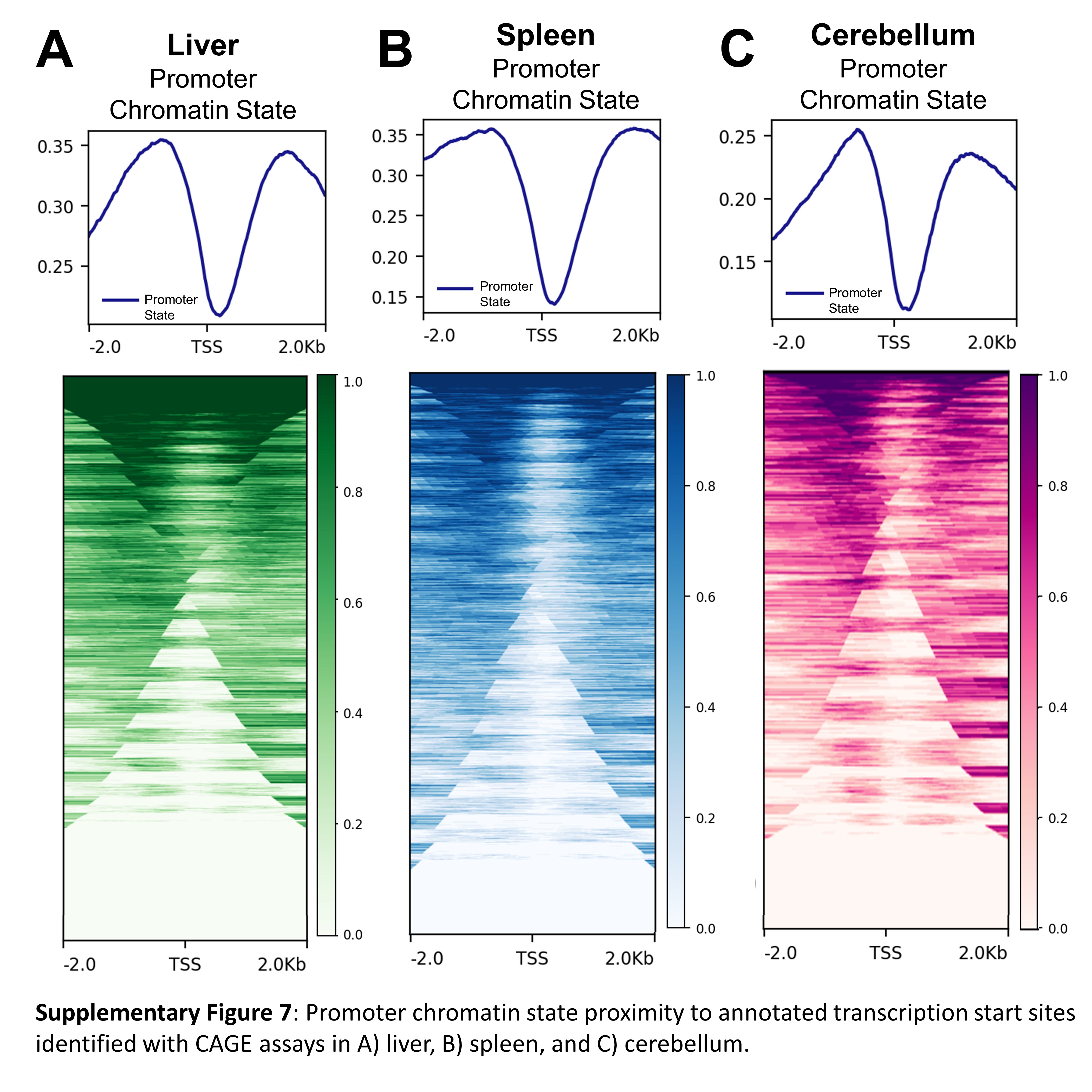

Supplement: Supplementary file 7 [file Image_7.JPEG]
