# Supplementary material for: Characterizing Genetic Regulatory Elements in Ovine Tissues
Source: Front Genet. 2021 May 20;12:628849. doi: 10.3389/fgene.2021.628849 (PMC8173140; doi:10.3389/fgene.2021.628849)
Supplement: Supplementary file 8 [file Table_1.docx]

**Supplementary Table 1.** ChIP-seq quality metrics for each library. In the sample label column, L, S, and C represent liver, spleen, and cerebellum, respectively. F and M followed by a number represent female and male animal numbers, respectively. NRF represents the non-redundant fraction of the library and FRiP represents the fraction of reads in peaks.

| **Tissue** | **Histone Mark** | **Sample** | **Unique Mapping %** | **Number of Uniquely Mapped Reads** | **NRF** | **Number of Peaks** | **FRiP** |
| --- | --- | --- | --- | --- | --- | --- | --- |
| **Liver** | **H3K4me3** | L_F1 | 76.26% | 91,892,045 | 0.43 | 10,648 | 0.01 |
|  |  | L_F2 | 75.41% | 51,369,218 | 0.27 | 9,062 | 0.01 |
|  |  | L_M1 | 74.35% | 27,240,631 | 0.82 | 10,745 | 0.01 |
|  |  | L_M2 | 74.04% | 37,575,540 | 0.83 | 11,376 | 0.01 |
|  | **H3K27ac** | L_F1 | 83.57% | 21,882,178 | 0.69 | 25,464 | 0.02 |
|  |  | L_F2 | 84.38% | 55,814,359 | 0.04 | 29,661 | 0.07 |
|  |  | L_M1 | 83.66% | 59,037,311 | 0.81 | 27,123 | 0.01 |
|  |  | L_M2 | 82.24% | 28,313,543 | 0.91 | 39,965 | 0.02 |
|  | **H3K4me1** | L_F1 | 80.26% | 31,870,135 | 0.65 | 40,632 | 0.14 |
|  |  | L_F2 | 84.06% | 74,783,676 | 0.62 | 30,153 | 0.15 |
|  |  | L_M1 | 82.98% | 33,532,918 | 0.90 | 60,874 | 0.25 |
|  |  | L_M2 | 80.56% | 54,530,540 | 0.91 | 59,655 | 0.26 |
|  | **H3K27me3** | L_F1 | 78.98% | 46,772,724 | 0.31 | 33,340 | 0.01 |
|  |  | L_M1 | 79.98% | 42,684,307 | 0.59 | 44,710 | 0.16 |
|  |  | L_M2 | 76.54% | 46,898,657 | 0.84 | 33,583 | 0.08 |
|  | **CTCF** | L_F1 | 62.41% | 69,727,193 | 0.01 | 29,893 | 0.07 |
|  |  | L_F2 | 70.58% | 40,958,273 | 0.03 | 30,762 | 0.03 |
|  |  | L_M1 | 78.55% | 41,219,481 | 0.07 | 16,036 | 0.14 |
|  |  | L_M2 | 81.50% | 39,388,145 | 0.19 | 29,378 | 0.13 |
| **Spleen** | **H3K4me3** | S_F1 | 73.77% | 25,536,400 | 0.27 | 16,936 | 0.01 |
|  |  | S_F2 | 77.72% | 25,364,432 | 0.78 | 10,381 | 0.01 |
|  |  | S_M1 | 78.84% | 34,624,081 | 0.79 | 12,640 | 0.01 |
|  |  | S_M2 | 77.63% | 28,406,550 | 0.80 | 13,601 | 0.01 |
|  | **H3K27ac** | S_F1 | 83.45% | 38,909,684 | 0.47 | 43,423 | 0.02 |
|  |  | S_F2 | 82.32% | 73,276,800 | 0.49 | 37,966 | 0.07 |
|  |  | S_M1 | 84.98% | 41,271,154 | 0.84 | 38,294 | 0.02 |
|  |  | S_M2 | 79.74% | 53,722,712 | 0.60 | 21,626 | 0.01 |
|  | **H3K4me1** | S_F1 | 75.54% | 46,268,769 | 0.04 | 24,923 | 0.28 |
|  |  | S_F2 | 82.21% | 91,786,917 | 0.06 | 39,769 | 0.15 |
|  |  | S_M1 | 77.83% | 49,634,682 | 0.42 | 22,742 | 0.09 |
|  |  | S_M2 | 77.08% | 37,868,373 | 0.06 | 30,762 | 0.25 |
|  | **H3K27me3** | S_F2 | 79.22% | 56,617,636 | 0.04 | 22,051 | 0.76 |
|  |  | S_M1 | 79.03% | 59,598,255 | 0.14 | 36,974 | 0.12 |
|  |  | S_M2 | 77.21% | 40,734,132 | 0.06 | 22,482 | 0.39 |
|  | **CTCF** | S_F2 | 77.92% | 73,893,274 | 0.02 | 33,599 | 0.16 |
|  |  | S_M1 | 76.93% | 43,394,622 | 0.18 | 29,006 | 0.03 |
|  |  | S_M2 | 74.08% | 41,908,970 | 0.06 | 22,482 | 0.06 |
| **Cerebellum** | **H3K4me3** | C_F1 | 80.47% | 38,211,509 | 0.86 | 16,542 | 0.01 |
|  |  | C_F2 | 80.14% | 57,349,641 | 0.81 | 16,116 | 0.01 |
|  |  | C_M1 | 78.49% | 24,280,387 | 0.95 | 21,463 | 0.01 |
|  |  | C_M2 | 79.31% | 37,236,584 | 0.89 | 13,524 | 0.01 |
|  | **H3K27ac** | C_F1 | 75.77% | 36,868,004 | 0.83 | 19,850 | 0.01 |
|  |  | C_F2 | 82.61% | 40,111,785 | 0.71 | 40,069 | 0.01 |
|  |  | C_M1 | 83.27% | 32,440,683 | 0.81 | 37,388 | 0.01 |
|  |  | C_M2 | 83.53% | 26,027,091 | 0.93 | 30,174 | 0.01 |
|  | **H3K4me1** | C_F1 | 77.99% | 31,415,709 | 0.94 | 47,600 | 0.10 |
|  |  | C_F2 | 82.35% | 90,253,571 | 0.89 | 58,760 | 0.26 |
|  |  | C_M1 | 80.24% | 38,566,786 | 0.92 | 51,334 | 0.36 |
|  |  | C_M2 | 79.97% | 57,547,807 | 0.95 | 49,369 | 0.26 |
|  | **H3K27me3** | C_F1 | 75.56% | 53,713,322 | 0.84 | 59,107 | 0.16 |
|  |  | C_F2 | 71.92% | 54,875,161 | 0.94 | 31,614 | 0.14 |
|  |  | C_M1 | 74.11% | 107,690,841 | 0.89 | 29,032 | 0.07 |
|  |  | C_M2 | 74.88% | 98,190,441 | 0.98 | 27,818 | 0.09 |
|  | **CTCF** | C_F1 | 75.56% | 53,713,322 | 0.25 | 22,405 | 0.01 |
|  |  | C_F2 | 75.51% | 39,423,803 | 0.59 | 20,455 | 0.01 |
|  |  | C_M1 | 74.71% | 28,256,482 | 0.74 | 30,170 | 0.01 |
|  |  | C_M2 | 75.00% | 34,533,188 | 0.95 | 31,945 | 0.01 |
