# Supplementary material for: Characterizing Genetic Regulatory Elements in Ovine Tissues
Source: Front Genet. 2021 May 20;12:628849. doi: 10.3389/fgene.2021.628849 (PMC8173140; doi:10.3389/fgene.2021.628849)
Supplement: Supplementary file 9 [file Table_2.docx]

**Supplementary Table 2.** DNA methylation quality metrics for each library. In the sample label column, L, S, and C represent liver, spleen, and cerebellum, respectively. F and M followed by a number represent female and male animal numbers, respectively.

| **Tissue** | **Sample** | **Total PE Reads** | **Validated PE Reads** | **Uniquely mapped PE Reads** | **Unmapped read pairs** | **Bases used for mapping** | **Bases uniquely mapped** | **mCG (%)** | **mCHG (%)** | **mCHH (%)** | **Mappability (%)** |
| --- | --- | --- | --- | --- | --- | --- | --- | --- | --- | --- | --- |
| **Liver** | L_F1 | 250802363 | 250773326 | 197117104 | 53656222 | 74105547825 | 58284149715 | 69.36 | 0.24 | 0.25 | 78.60 |
|  | L_F2 | 305118183 | 305106713 | 234081202 | 71025511 | 90938436967 | 69802293190 | 69.75 | 0.25 | 0.25 | 76.72 |
|  | L_M1 | 272377520 | 272367391 | 225870709 | 46496682 | 81189499413 | 67357790499 | 71.40 | 0.24 | 0.24 | 82.93 |
|  | L_M2 | 251382724 | 251374337 | 194073342 | 57300995 | 74923337158 | 57872016595 | 66.72 | 0.24 | 0.23 | 77.20 |
| **Spleen** | S_F1 | 218460634 | 218451691 | 177966878 | 40484813 | 65111181250 | 53071678722 | 75.68 | 0.25 | 0.26 | 81.47 |
|  | S_F2 | 201358535 | 201273605 | 168451070 | 32822535 | 58730553503 | 49198143343 | 77.69 | 0.21 | 0.29 | 83.69 |
|  | S_M1 | 271369128 | 271357982 | 222096734 | 49261248 | 80874164415 | 66226685025 | 76.44 | 0.25 | 0.25 | 81.85 |
|  | S_M2 | 213583077 | 213573474 | 177063138 | 36510336 | 63652634020 | 52796851913 | 75.80 | 0.24 | 0.24 | 82.91 |
| **Cerebellum** | C_F1 | 217567096 | 217558899 | 183491333 | 34067566 | 64855584915 | 54720375734 | 79.50 | 1.60 | 2.01 | 84.34 |
|  | C_F2 | 301162248 | 301152811 | 254518260 | 46634551 | 89774041124 | 75900622947 | 81.06 | 1.60 | 2.03 | 84.51 |
|  | C_M1 | 210546324 | 210540044 | 178412725 | 32127319 | 62755392823 | 53201279166 | 80.33 | 1.88 | 2.38 | 84.74 |
|  | C_M2 | 256873526 | 256864142 | 214165602 | 42698540 | 76567651870 | 63867562503 | 80.39 | 1.54 | 1.99 | 83.38 |
|  |  |  |  |  |  |  |  |  |  |  |  |
| **Average per tissue** | Liver | 1,079,680,790 | 1,079,621,767 | 851,142,357 | 228,479,410 | 321,156,821,363 | 253,316,249,999 | 69.31 | 0.24 | 0.24 | 78.86 |
|  | Spleen | 904,771,374 | 904,656,752 | 745,577,820 | 159,078,932 | 268,368,533,188 | 221,293,359,003 | 76.40 | 0.24 | 0.26 | 82.48 |
|  | Cerebellum | 986,149,194 | 986,115,896 | 830,587,920 | 155,527,976 | 293,952,670,732 | 247,689,840,350 | 80.32 | 1.65 | 2.10 | 84.24 |
