# Supplementary material for: Characterizing Genetic Regulatory Elements in Ovine Tissues
Source: Front Genet. 2021 May 20;12:628849. doi: 10.3389/fgene.2021.628849 (PMC8173140; doi:10.3389/fgene.2021.628849)
Supplement: Supplementary file 10 [file Table_3.docx]

**Supplementary Table 3.** Mean number of peaks called per histone mark in each tissue compared to the number of peaks called in these histone marks in other published studies.

| **Histone Mark** | **Sheep Liver*** | **Sheep Spleen*** | **Sheep Cerebellum*** | **Sheep Adipose†** | **Cattle Liver⁑** | **Cattle Muscle‡** | **Cattle Rumen Epitheliumᶲ** | **Human Liver⁑** | **Mouse Liver⁑** |
| --- | --- | --- | --- | --- | --- | --- | --- | --- | --- |
| **H3K4me3** | **10,458** | **13,389** | **16,911** | **16,098** | **13,552** | **11,924** | **30,348** | **12,453** | **14,571** |
| **H3K27ac** | **30,553** | **35,327** | **35,877** | **35,622** | **52,481** | **-** | **43,726** | **50,143** | **36,559** |
| **H3K4me1** | **47,828** | **33,931** | **51,766** | **-** | **-** | **-** | **50,738** | **-** | **-** |
| **H3K27me3** | **39,162** | **29,939** | **36,893** | **31,942** | **-** | **-** | **29,456** | **-** | **-** |
| **CTCF** | **26,517** | **28,362** | **26,244** | **-** | **-** | **-** | **-** | **-** | **-** |

*This study, †Naval-Sanchez et al., 2018; ⁑Villar et al., 2015; ‡Zhao et al., 2015; ᶲFang et al., 2019
